# Supplementary material for: Help or Hindrance? The Alcohol Industry and Alcohol Control in Portugal
Source: Int J Environ Res Public Health. 2019 Nov 18;16(22):4554. doi: 10.3390/ijerph16224554 (PMC6888387; doi:10.3390/ijerph16224554)
Supplement: Supplementary file 1 [file ijerph-16-04554-s001.pdf]

Table S1 - Justification of the selection of industry actors and initiatives funded by the industry.

Trade association/ initiative	Justification for the inclusion
**Organização interprofissional do vinho de Portugal**	It is a private non-profit representative of eight wine trade associations and created by the Ministry of Agriculture. It lists the Ministry of Agriculture and Sea and the Directorate-General of Agriculture and Rural development as partners. It is also the creator of the organisation "Beba com cabeça".<br><br>It represents the following trade associations: ACIBEV - Associação de Vinhos e Espirituosas de Portugal, ANCEVE - Associação Nacional dos Comerciantes, e Exportadores de Vinhos e Bebidas Espirituosas, AND - Associação Nacional de Destiladores de Produtos e Sub-Produtos, ANDOVI - Associação Nacional das Denominações de Origem Vitivinícolas, CAP - Confederação dos Agricultores de Portugal, FENADEGAS - Federação Nacional de Adegas Cooperativas, FENAVI - Federação Nacional de Viticultores Independentes, FEVIPOR - Federação de Viticultores de Portugal.<br><br>Overall it represents over 370 national groups.
**Associação de vinhos e espirituosas de Portugal**	It accounts for 40% of the national market of wine according to their website(36). It represents the following 93 producers:<br><br>A.S.L- Tomé- Sociedade Vinícola, Lda.; Adega do Cantor Sociedade de Viniviticultura, Lda.; Alfredo Dias da Silva & Filhos, Lda.; Aliança Vinhos de Portugal; António Bernardino Paulo da Silva; António Francisco Bonifácio & Filhos, Lda.; Aveleda S.A.; Bacalhôa - Vinhos de Portugal S.A., Lda.; BCH – Comércio de Vinhos, S.A.; Bebilusa – Indústria e Comércio de Bebidas, Lda.; Bernardinos & Carvalho, S.A.; CARM – Casa Agrícola Roboredo Madeira, S.A.; Carvalho, Ribeiro & Ferreira, Lda.; Casa Agrícola Alexandre Relvas, Lda.; Casa Ermelinda Freitas – Vinhos, Lda.; Casal Branco – Sociedade de Vinhos, S.A.; Cave Central da Bairrada, S.A; Caves Arcos do Rei, Lda.; Caves Campelo, S.A; Caves da Cerca, S.A; Caves da Raposeira, S.A; Caves d'alagoa – Sociedade Agro-industrial, Lda.; Caves do Monte Vinhos, S.A; Caves São João- Sociedade dos Vinhos Unidos, Lda.; Cavipor – Vinhos de Portugal, S.A; COMTEMP – Companhia de Temperos, Lda.; Destilaria Levira, Lda.; Duorum Vinhos, S.A; Enoport United Wines S.A.; Enolavor, Agro-Turismo, S.A.; Esporão S.A; Falua - Sociedade de Vinhos, S.A; Fereira Gomes & Filhos, Lda.; Ferreira Malaquias, Lda.; Frutóbios, Lda.; Goanvi Bottling, Lda; Herdade da Candeeira Unipessoal, Lda.; Herdade da Comporta S.A.; Herdade da Vigia

	–Sociedade Vitivinícola Unipesso; João Portugal Vinhos S.A.; José Maria da Fonseca Vinhos, S.A; Julia Kemper Wines S.A; Lima & Smith, Lda.; LMH Wines Lda.; MAGNUM Carlos Lucas Lda.; Manuel da Costa Carvalho Lima & Filhos, Lda.; Marcolino Sebo Wines & Oils, Lda.; Mendes Gonçalves S.A.; Monte da Capela, Lda.; Murças, S.A.; Muxagat Vinhos, Lda.; Paço das Cortes, Lda.; Parras Wines Unipessoal, Lda.; QBL – Quinta do Barranco Longo, Unipessoal Lda.; Quinta da Alorna Vinhos, Lda.; Quinta da Lixa – Sociedade Agrícola, Lda.; Quinta das Arcas – Sociedade Agrícola, Lda.; Quinta das Tecedeiras – Sociedade Vitivinícola, Lda.; Quinta de Foz de Arouce; Quinta do Crasto, S.A.; Quinta do Gradil – Sociedade Vitivinívola, S.A.; Quinta do Morgado da Torre, Lda.; Quinta dos Vales- Agricultura e Turismo, S.A.; Quinta Nova da Nossa Senhora do Carlo, S.A.; Quinta Vale D. Maria Vinhos, S.A.; Roquevale, S.A.; Santos & Santos; SIVIPA – Sociedade Vinícola de Palmela, S.A.; Sociedade Agrícola Boas Quintas, Lda.; Sociedade Agrícola D. Diniz, S.A.; Sociedade Agrícola da Fonte Coberta,Lda.; SociedadeAgrícola da Herdade das Mouras de Arraiolas, S.A.; Sociedade Agrícola da Romaneira, S.A.; Sociedade Agrícola Comercial do Varosa, S.A.; Sociedade Agrícola Quinta de Soutelos, Lda.; Sociedade de Vinhos Victor Matos II, S.A.; Sociedade de Vinhos Borges, S.A.; Sogrape Vinhos, S.A.; Sovibor – Sociedade de Vinhos de Borba, Lda.; Tapada das Gaeiras Unipes; Transwine Production Lda.; Venâncio da Costa Lima – SUCS, Lda.; Viborel Distribuição, S.A.; Vinorte – Vinícola do Norte, Lda.; Wine ventures – Quinta da Romeira, S.A.; Xavier Santana – SUCS, Lda.
**Sociedade Central de Cervejas**	It represented 43.6% of the national beer market in 2013 according to their website(37).
**Associação de Cervejeiros de Portugal**	It represents 14 producers/retailers (including 2 brands that represent over 90% of the domestic trade of beer according to a market study (38))<br><br>ECM – Empresa de Cervejas da Madeira, Sociedade Unipessoal, Lda.; Font Salem – Portugal, SA; SCC-Sociedade Central de Cervejas e Bebidas, SA; SuperBock Group; Essência d'Alma Lda; Happy evasion; Os trªes cervejeiros, Lda.; Praxis Cervejas de Coimbra Lda.; Fábrica de Cervejas Portuense, PRHM TRT Lda.; Post Scriptum Brewery, Unipessoal, Lda.; Faustino – Microvervejeira, Lda.; Poder do Lúpulo - Cervejeiros, Lda.; Beam Park, Lda.; Cervejaria Crafters, Lda.

**Associação Nacional de Empresas de Bebidas Espirituosas**	It represents the following 33 producers:<br><br>Bacardi; DIAGEO; Licor Beirão; Pernod Ricard; Vigne – Gin de France; Decanter garrafeira; Gold Grail; Aguardente Medronho; Dom Cristina; Ginja sem rival; ADAMVS; Friends Gin Premium; Sharish Gin; Limontejo; Vila das Rainhas; Oficina de Espíritos; Premium GIN NAO; IK; W Valley; Gin Loers; Neighbours premium gin; Licores Ezequiel; Licor de Óbidos Mariquinhas; Manuel Rainha; William Hinton Rum; Bagamel; Licor Arradidine; Louriana XO; MVP Gin; Gint Premium Spirits; Ginja de Óbidos Oppidum; amarguinha: IN Separable Craft Gin.<br><br>Also funded a non-profit.
**“Beba com cabeça” initiative**	Non-profit organization funded by the “Organização Interprofissional do Vinho de Portugal”, previously included.
**“100% Cool”**	Non-profit organization funded by the “Associação Nacional de Empresas de Bebidas Espirituosas”, previously included.

Table S2 - Sources of information used to extract publicly-available material. Adapted from Mialon et al. (22)

Nature of the source of the information		Sources of information
**Trade associations**	Associação nacional de empresas de bebidas espirituosas	http://anebe.pt/home/
	Organização Interprofissional do Vinho de Portugal	https://www.viniportugal.pt/OSector<br>https://twitter.com/wopbr?lang=pt
	Sociedade central de cervejas	http://www.centralcervejas.pt/consumoresponsavel/consumo-responsavel.aspx
	Associação de vinhos e espirituosas de Portugal	https://www.acibev.pt/
	Associação dos cervejeiros de Portugal	https://www.cervejeirosdeportugal.pt/
**AI funded organizations**	"Beba com cabeça"	http://bebacomcabeca.pt/<br>https://www.facebook.com/bebacomcabeca
	"100 % cool"	http://www.100porcentocool.pt/<br>https://www.facebook.com/100porcentocool
	"Menores nem uma gota"	https://menoresnemumagota.pt/
**Government material**	Portuguese Parliament	https://www.parlamento.pt/
	Ministry of Health	https://www.sns.gov.pt/
	General-Directorate of Health	https://www.dgs.pt/

	General-Directorate for intervention on addictive behaviours and dependencies	http://www.sicad.pt/
**Media**	Correio da Manhã	https://www.cmjornal.pt/
	Público	https://www.publico.pt/
	Jornal de Notícias	https://www.jn.pt/
	Notícias ao Minuto	http://www.noticiasaominuto.com/
	Expresso	https://expresso.pt/
**Universities**	Universidade de Lisboa	https://www.ulisboa.pt/en/
	Universidade Nova de Lisboa	https://www.unl.pt/
	Universidade de Coimbra	http://www.uc.pt/
	Universidade do Minho	https://www.uminho.pt/PT
	Universidade da Beira Interior	https://www.ubi.pt/
	Universidade do Algarve	https://www.ualg.pt/pt
	Universidade do Porto	

Table S3 - Framework of the instrumental strategies used by the tobacco industry, extracted and adapted from Ulucanlar et al.(12) and merged with the framework from Savell et al.(11).

**Instrumental strategies**	**Practices**

**Coalition management**	Constituency recruitment	Internal
		Establish relationships with key opinion leaders and health organisations
		Seek involvement in the community
		Establish relationship with the media
	Constituency fabrication	
	Constituency fragmentation	
**Information management**	Production	
	Amplification	
	Suppression	
	Credibility	
**Direct involvement and influence in policy**	Indirect access	
	Incentives	
	Threats	
	Actor in the decision making process	

**Legal actions**	Use legal action (or the threat thereof) against public policies or opponents
	Influence the development of trade and investment agreements/ shape international law

Table S1 - Framework of the discursive strategies used by the tobacco industry, extracted and adapted from Ulucanlar et al.(12) and merged with the framework from Savell et al. (11).

Discursive strategy	Domain	Argument
**Unanticipated costs to the economy**	The economy	The policy will lead to lost sales/jobs
		The policy will lead to lost/unreliable tax revenue
		The regulation is discriminatory/ will not affect all producers equally
	Law enforcement	Policy will increase illicit trade
		Policy will criminalise the public
	The law	Breach of intellectual property laws and trade agreements
		Public body acting beyond jurisdiction
	Politics/governance	Government is anti-free enterprise and interferes with a free market economy
		The government is unreasonable and unaccountable
		Policymakers and public health actors act authoritarian
	Social justice	Policy is unfair to drinkers
		Policy is regressive
**Unintended benefits to undeserving groups**		Non-legal business will increase and smugglers will profit
		Big business will profit
**Unintended costs to public health**		Negative public health consequences
**Intended public health benefits**	There is not good enough evidence	

	Policy will not work
	Policy is not needed because the industry is responsible and the existing regulation is satisfactory
	Policy is not needed because the industry is responsible and the existing regulation is satisfactory just needs better inforcement
**Expected industry costs**	The policy will lead to a decrease in sales and jobs in both manufacturers and associated industries
	The regulation is discriminatory and will not affect all producers equally
	Cost and time of compliance will be high
**Others**	Argue the case for self-regulation by the industry, education and an individual-level approach focusing on the drinker
	Collaboration with the industry would be beneficial

Table S5 – Data extracted and coded. Table adapted from Mialon et al.(19)

Reference in manuscript	Source	Strategy	Practice /argument	Data coded	Notes	Website URL
**A1**	ANEBE	Instrumental	Relationships with leaders/health organisations	"A ANEBE, como organização fundadora e membro atual da comissão executiva do Fórum Nacional Álcool e Saúde (FNAS), partilha objetivos de missão com a comunidade da saúde. "		http://anebe.pt/home/ (accessed 15th May 2019)
**A2**	ANEBE	Discursive	Self-regulation/education	"Os projetos da ANEBE, comprometidos com as metas nacionais do PNRCAD2020 para a redução do consumo nocivo, contribuem ativamente para o reforço da prevenção e educação para o consumo responsável, moderado e inteligente."		http://anebe.pt/home/ (accessed 15th May 2019)
**A3**	ANEBE	Instrumental	Internal constituency recruitment	"Fundada no ano de 2000, a ANEBE é a plataforma nacional da indústria de bebidas espirituosas e tem a missão primordial de dar voz coletiva a um setor que representa a maior exportação de produtos agro-alimentares da União Europeia"		http://anebe.pt/home/ (accessed 15th May 2019)
**A4**	ANEBE	Discursive	Self-regulation/education	"Disfrute de um consumo inteligente"		http://anebe.pt/home/ (accessed 15th May 2019)
**A5**	ANEBE	Discursive	Self-regulation/education	"Os códigos de mensagem e comunicação em torno do próprio sublinham o mérito pessoal e social do condutor, que a cada noite é responsável pela segurança do grupo, e, portanto, destacam o valor social deste comportamento. Ser responsável é Cool."		http://anebe.pt/consumo_inteligente/ (Accessed 15th May 2019)

**A6**	ANEBE	Instrumental	Community involvement	"É particularmente meritória a sólida cooperação com as autoridades de segurança, a colaboração com parceiros privados,…"		http://anebe.pt/consumo_inteligente/ (Accessed 15th May 2019)
**A7**	ANEBE	Instrumental	Community involvement	"… seu 15º Aniversário no ano de 2017 é o contexto indicado para revalidar um novo ciclo de pedagogia ativa junto da sociedade portuguesa."		http://anebe.pt/consumo_inteligente/ (Accessed 15th May 2019)
**A8**	ANEBE	Discursive	Self-regulation/education	"...contribuir para a consolidação de um padrão de consumo mais inteligente, moderado e responsável."		http://anebe.pt/consumo_inteligente/ (Accessed 15th May 2019)
**A9**	ANEBE	Discursive	Self-regulation/education	"Este renovado portal de informação sobre o consumo de bebidas aloólicas, lançado em 2008 pela ANEBE é uma aposta na sensibilização dos consumidores para uma atitude inteligente de consumo moderado e responsável. grande objectivo do site bebacomcabeca.pt é providenciar informação credível e consistente de forma a dissipar as falsas verdades e preconceitos em torno do álcool, promovendo ao mesmo tempo o conceito de unidade de bebida padrão (UBP). A funcionalidade prática de relevo deste site é a possibilidade de o consumidor ter sempre disponível um alcooladora que lhe providencia a taxa de alcoolémia estimada, o nº de calorias ingeridas e a quantidade de álcool puro em cada bebida."		http://anebe.pt/consumo_inteligente/ (Accessed 15th May 2019)

**A10**	ANEBE	Discursive	Existing regulation is satisfactory	"Conformidade do Conteúdo da Comunicação Comercial, bem como da Conformidade da Ética da Frase Educacional, tendo em consideração o universo total de anúncios monitorizados e considerando a sua re-análise, quando necessário, a Monitorização durante o ano de 2016 teve 100% de Conformidade Ética e Legal."		http://anebe.pt/consumo_inteligente/ (Accessed 15th May 2019)
**A11**	ANEBE	Instrumental	Community involvement	"O movimento #menoresnemumagota tem a missão de mobilizar a sociedade portuguesa em torno das metas e objetivos nacionais inscritos no pnrcad2020 (plano nacional para a redução de comportamentos aditivos) que visam a redução do consumo de bebidas alcoólicas por menores, cumprindo assim o normativo legal previsto no decreto-lei nº 106/2015 que proíbe a venda e consumo de bebidas alcoólicas em locais públicos e em locais abertos ao público a menores de 18 anos de idade. Entre as prioridades do iii ciclo de ação do fórum nacional álcool e saúde (2017-2020), a redução do consumo entre as jovens gerações assume especial relevância."		http://anebe.pt/consumo_inteligente/ (Accessed 15th May 2019)
**A12**	ANEBE	Instrumental	Community involvement	"O quadro de multi-parcerias, públicas e privadas, em que a ANEBE desenvolve os seus programas de responsabilidade social é demonstrativo da amplitude estratégica da sua ação e potenciador de resultados e impactos positivos na sociedade portuguesa."		http://anebe.pt/consumo_inteligente/ (Accessed 15th May 2019)
**A13**	ANEBE	Instrumental	Internal constituency recruitment	A atividade da ANEBE rege-se pelos princípios da cooperação e colaboração entre membros associados, valorizando a voz coesa e unida de um setor cada vez mais relevante para a economia nacional.		http://anebe.pt/associados/ (accessed 15th May 2019)
**A14**	ANEBE	Discursive	Importance of the industry to the economy	"A indústria representa 679 milhões em vendas (...) Relevante para a economia, 254 milhões € em contribuições para o		http://anebe.pt/setor/

				Estado. A indústria emprega 5940 direta e indiretamente em Portugal."		(accessed 15th May 2019)
**A15**	ANEBE	Discursive	Self-regulation/education	"ANEBE líder na promoção do consumo inteligente; responsável e auto-regulada."		http://anebe.pt/setor/ (accessed 15th May 2019)
**A16**	ANEBE	Discursive	Other/culture	"Tradicional e nacional."		http://anebe.pt/setor/ (accessed 15th May 2019)
**A17**	ANEBE	Discursive	Other/pattern of consumption	"Consumo per capita espirituosas 1L vs 2.6L média EU (litros de álcool puro)."		http://anebe.pt/setor/ (accessed 15th May 2019)
**A18**	ANEBE	Discursive	Discriminatory among the producers	"11% do mercado suporta 54.5% da receita total do IABA…"		http://anebe.pt/setor/ (accessed 15th May 2019)
**A19**	ANEBE	Discursive	Importance of the industry to the economy	"A matriz de missão da ANEBE prioriza a valorização do contributo e impacto da nossa indústria na economia nacional…"		http://anebe.pt/anebe/ (accessed 20th May 2019)
**A20**	ANEBE	Discursive	Self-regulation/education	"A matriz de missão da ANEBE prioriza (…) a promoção de padrões de consumo inteligente e informação ao consumidor."		http://anebe.pt/anebe/ (accessed 20th May 2019)
**A21**	ANEBE	Instrumental	Internal constituency recruitment	"… a Associação Nacional de Empresas e bebidas espirituosas reúne as principais empresas de bebidas espirituosas em Portugal"		http://anebe.pt/anebe/

						(accessed 20th May 2019)
**A22**	ANEBE	Discursive	Lost/unreliable tax revenue	"...O sector debate-se com o incremental aumento da carga fiscal, que encara como destruidor de valor e um elemento desincentivador desse mesmo investimento(...) é uma pressão distorciva sobre o mercado e destruidora de valor para a indústria. Acresce que também é ineficiente."		http://www.anebe.pt/noticias/page/2/ (accessed 23rd May 2019)
**A23**	ANEBE	Instrumental	Amplification of information	"...insustentável equação do IABA (...) Consideramos ser a fórmula que melhor rentabilizaria o atual momento da economia(...) permitiria às autoridades portuguesas arrecadar receita além dos 200 milhões de euros com o IABA anualmente."		http://www.anebe.pt/noticias/page/2/ (accessed 23rd May 2019)
**A24**	ANEBE	Discursive	Lost/unreliable tax revenue	" No entender da indústria, a estabilidade fiscal é o valor chave para o relançamento da competitividade deste sector, no sentido de estimular o crescimento do emprego, das exportações e do investimento, por via da previsibilidade sobre o ambiente fiscal. Até porque, apesar da contração sentida nos últimos anos, a indústria das bebidas espirituosas tem contribuído para o aumento das exportações portuguesas. "		http://www.anebe.pt/noticias/page/2/ (accessed 23rd May 2019)
**A25**	ANEBE	Discursive	Importance of the industry to the economy	" Responsável pela manutenção de 159 postos de trabalho na Região, a produção de bebidas espirituosas está diretamente ligada ao setor agrícola madeirense, contribuíndo para o escoamento de fruta regional que é utilizada na produção dos licores. Esta atividade tem assumido um peso cada vez mais significativo na economia regional"		http://www.anebe.pt/noticias/page/2/ (accessed 23rd May 2019)

**A26**	ANEBE	Instrumental	Community involvement	" A mytaxi, empresa líder europeia em serviços digitais de reserva de táxis, associa-se como parceiro estratégico do programa "100% COOL: Edição 15 Anos", com o objetivo de promover em conjunto com a ANEBE (Associação Nacional de Empresas de Bebidas Espirituosas) a taxa 0% de álcool ao volante entre os condutores mais jovens (...) em operações que a ANEBE realiza em parceira com a PSP e GNR há já 15 anos por todo o território nacional e que tem marcado várias gerações de condutores portugueses."		http://www.anebe.pt/noticias/page/2/ (accessed 23rd May 2019)
**A27**	ANEBE	Instrumental	Community involvement	" Em 2018, esta iniciativa que marcou inúmeras gerações, volta à estrada e às redes sociais com mais força, com novos parceiros públicos e privados e com um compromisso mais ambicioso de reduzir a relação do álcool com a condução"		http://www.anebe.pt/noticias/page/2/ (accessed 23rd May 2019)
**A28**	ANEBE	Discursive	Illicit trade	"Quanto maior for o diferencial da taxa face a Espanha, mais apelativas se tornam as condutas fraudulentas ou a mera preferência do consumidor pela compra destes produtos do outro lado da fronteira."		http://www.anebe.pt/noticias/ (accessed 23rd May 2019)
**A29**	ANEBE	Instrumental	Amplification of information	"A Associação Nacional de Empresas de Bebidas Espirituosas (ANEBE) quer que o Governo congele o imposto aplicável ao setor em 2019 e promete que isso não resultará numa quebra de receitas, mas sim num crescimento de 3,6%. "		http://www.anebe.pt/noticias/ (accessed 23rd May 2019)
**A30**	ANEBE	Instrumental	Relationships with leaders/health organisations	"A iniciativa resulta de um protocolo entre a Associação Nacional de Empresas de Bebidas Espirituosas (ANEBE), o Rock In Rio e a Divisão de Intervenção nos Comportamentos Aditivos e nas Dependências (DICAD) da ARS Lisboa, que é assinado hoje, na cidade do rock"		http://www.anebe.pt/noticias/ (accessed 23rd May 2019)

**A31**	ANEBE	Instrumental	Relationships with leaders/health organisations	"Neste sentido, a Divisão de Intervenção em Comportamentos Aditivos e Dependências da Administração Regional de Saúde de Lisboa e Vale do Tejo vai desenvolver este ano uma ação em conjunto com a Associação Nacional de Empresas de Bebidas Espirituosas "18 +"."		http://www.anebe.pt/noticias/ (accessed 23rd May 2019)
**A32**	ANEBE	Discursive	Self-regulation/education	"Neste sentido, a Divisão de Intervenção em Comportamentos Aditivos e Dependências da Administração Regional de Saúde de Lisboa e Vale do Tejo vai desenvolver este ano uma ação em conjunto com a Associação Nacional de Empresas de Bebidas Espirituosas (...) formou cerca de 80 jovens universitários, que no recinto irão colocar as pulseiras 18 + BEBA COM CABEÇA sensibilizando os jovens maiores de 18 para um consumo inteligente."		http://www.anebe.pt/noticias/ (accessed 23rd May 2019)
**A33**	ANEBE	Instrumental	Relationships with leaders/health organisations	"Durante a visita à Madeira Rui Duarte visitou o Engenho Novo da Calheta, a J.Faria, o secretário regional da Saúde, e vai ainda promover uma campanha alertando para a necessidade do consumo moderado e responsável de bebidas alcoólicas integradas no Fórum Regional Álcool e Saúde."		http://www.anebe.pt/noticias/ (accessed 23rd May 2019)
**A34**	ANEBE	Discursive	Importance of the industry to the economy	"Com uma fiscalidade mais favorável poderíamos ter a industria com maior peso ainda na economia nacional, porque vamos crescer até 2021 com taxas anuais acima dos 2,5 a 3 por cento, o que significa que crescendo a um ritmo superior à inflacção este sector é cada vez mais o contribuinte líquido para o crescimento económico nacional"		http://www.anebe.pt/noticias/ (accessed 23rd May 2019)
**A35**	ANEBE	Discursive	Interferes with a free market economy	"A carga fiscal que recai sobre as bebidas espirituosas é um fator de perda de competitividade e inibe o crescimento do negócio, aponta a Associação Nacional de Empresas de Bebidas Espirituosas (ANEBE)."		http://www.anebe.pt/noticias/ (accessed 23rd May 2019)

**A36**	ANEBE	Discursive	Discriminatory among the producers	"Nos últimos seis anos, o IABA (imposto sobre o álcool e as bebidas alcoólicas) aumentou mais de 30%, refere o secretário-geral da ANEBE, Rui Duarte, salientando que as espirituosas representam apenas 11% do consumo de bebidas alcoólicas, mas suportam quase 60% da receita deste Imposto Especial sobre o Consumo"		http://www.anebe.pt/noticias/ (accessed 23rd May 2019)
**A37**	ANEBE	Instrumental	Production of information	" A pedido da ANEBE, a consultora EY fez um estudo sobre a tributação das bebidas espirituosas em Portugal (abril de 2018) em que concluiu que a manutenção do IABA "permitirá a arrecadação de mais 12 milhões de euros em 2019". "		http://www.anebe.pt/noticias/ (accessed 23rd May 2019)
**A38**	ANEBE	Instrumental	Amplification of information	"A ANEBE, tendo em vista o Orçamento de Estado 2019, faz chegar uma proposta alternativa às autoridades portuguesas que considera "win-win" para 2019."		http://www.anebe.pt/noticias/ (accessed 23rd May 2019)
**A39**	ANEBE	Instrumental	Amplification of information	"'standstill' do IABA para 2019 represente um acréscimo de receita na ordem dos 12 milhões de euros", podendo ainda "abrir caminho para um quadro fiscal mais estável e eficiente, e, por conseguinte, mais amigo do investimento, do emprego e das exportações portuguesas""		http://www.anebe.pt/noticias/ (accessed 23rd May 2019)
**A40**	Associação de vinhos e espirituosas de Portugal	Instrumental	Relationships with leaders/health organisations	"No dia 11 de abril, uma delegação da ACIBEV, composta pelo Presidente George Sandeman, Ana Isabel Alves e Márcia Azevedo Rocha, participou no Fórum Nacional Álcool e Saúde, que se realizou em Loures. "		https://www.acibev.pt/pt/noticias/info/acibev-representa-setor-do-vinho-no-fnas_615/ (accessed 28th May 2019)

**A41**	Associação de vinhos e espirituosas de Portugal	Discursive	Self-regulation/education	"... a ACIBEV esteve presente em mais uma edição da Essência do Vinho, no Porto, com o Programa "Vinho com Moderação", para promover o consumo responsável como uma norma cultural/social de modo a prevenir e reduzir o abuso e os malefícios relacionados com o álcool."		https://www.acibev.pt/pt/noticias/info/acibev-presente-em-mais-uma-essencia-do-vinho_574/ (accessed 28th May 2019)
**A42**	Associação de vinhos e espirituosas de Portugal	Instrumental	Community involvement	"No sentido de sensibilizar profissionais do setor vitivinícola e, em particular, do setor de restauração e hotelaria, para as responsabilidades inerentes à venda de bebidas alcoólicas, a ACIBEV apresentou também o seu projeto "O Serviço Responsável faz o Negócio Sustentável", através da distribuição de folhetos e manuais de formação."		https://www.acibev.pt/pt/noticias/info/acibev-presente-em-mais-uma-essencia-do-vinho_574/ (accessed 28th May 2019)
**A43**	Associação de vinhos e espirituosas de Portugal	Discursive	Other/war on alcohol	"A Irlanda está em polvorosa. Uma lei recente obriga as garrafas a conterem alertas sobre os perigos para a saúde provenientes do consumo regular de álcool. " "É um precedente muito negativo e o receio é que possa ter efeito de contágio em toda a Europa", teme, em declarações à NM, Ana Isabel Alves, secretária-geral da Associação de Vinhos e Espirituosas de Portugal."		https://www.acibev.pt/pt/noticias/info/guerra-ao-alcoolismo-faz-ricochete-no-vinho_537/ (accessed 28th May 2019), redirected to https://www.noticiasmagazine.pt/2019/guerra-ao-alcoolismo-faz-ricochete-no-

						vinho/?fbclid=IwAR07O7vCFDblF2BL7kk0mtAY-GvhtzXQCMC3zKi0c96mdMZuXXPTXrqvOt4
**A44**	Associação de vinhos e espirituosas de Portugal	Instrumental	Relationships with leaders/health organisations	" A ACIBEV esteve presente no Fórum Anual dos Vinhos de Portugal, em Coimbra, no Convento de S. Francisco. Um dos painéis de destaque deste fórum foi sobre Vinho e Saúde, com as apresentações do Dr. Manuel Cardoso e do Dr. Nuno Abreu."		https://www.acibev.pt/pt/noticias/info/acibev-no-forum-anual-da-viniportugal_470/ (accessed 28th May 2019)
**A45**	Associação de vinhos e espirituosas de Portugal	Instrumental	Relationships with leaders/health organisations	"O evento realizou-se nos dias 23 e 24 de novembro (…) onde participou o Secretário de Estado da Agricultura, Luís Vieira."		https://www.acibev.pt/pt/noticias/info/acibev-na-abertura-da-dao-capital_469/ (accessed 28th May 2019)

**A46**	Associação de vinhos e espirituosas de Portugal	Instrumental	Internal constituency recruitment	"No dia 13 de novembro, George Sandeman, Presidente da ACIBEV e do WIM, e Ana Isabel Alves, Secretária-Geral da ACIBEV, estiveram presentes na Assembleia Geral da Associação Wine in Moderation."		https://www.acibev.pt/pt/noticias/info/acibev-na-assembleia-geral-do-wine-in-moderation_466/ (accessed 28th May 2019)
**A47**	Associação de vinhos e espirituosas de Portugal	Discursive	Self-regulation/education	"No dia 13 de novembro, George Sandeman, Presidente da ACIBEV e do WIM, e Ana Isabel Alves, Secretária-Geral da ACIBEV, estiveram presentes na Assembleia Geral da Associação Wine in Moderation. Partiparam ainda nos dois workshops, um científico sobre os últimos estudos relacionados com o Vinho e Saúde e outro sobre o papel da autorregulação no futuro da comunicação comercial."		https://www.acibev.pt/pt/noticias/info/acibev-na-assembleia-geral-do-wine-in-moderation_466/ (accessed 28th May 2019)
**A48**	Associação de vinhos e espirituosas de Portugal	Instrumental	Community involvement	"ACIBEV marca presença na feira do cavalo da Golegã."		https://www.acibev.pt/pt/noticias/info/acibev-marca-presenca-na-feira-do-cavalo-da-golega_465/ (accesssed 28th May 2019)
**A49**	Associação de vinhos e espiritu	Discursive	Self-regulation/education	"À saída do evento, a ACIBEV convidou os consumidores a realizar o teste de alcoolemia, antes de decidirem se vão conduzir, e distribui informação sobre o consumo moderado e responsável de bebidas alcoólicas."		https://www.acibev.pt/pt/noticias/info/acibev-presente-no-encontro-com-o-vinho-e-

	osas de Portugal					sabores-2018_460/ (accessed 28th May 2019)
**A50**	Associação de vinhos e espirituosas de Portugal	Instrumental	Community involvement	"À saída de um dos maiores eventos de vinho do País, a ACIBEV convida os visitantes a fazerem o teste de alcoolemia antes de decidirem conduzir e distribui diversa informação sobre o consumo moderado e responsável de bebidas alcoólicas."		https://www.acibev.pt/pt/noticias/info/acibev-volta-a-marcar-presenca-no-grandes-escolhas-vinhos-sabores_458/ (accessed 28th May 2019)
**A51**	Associação de vinhos e espirituosas de Portugal	Discursive	Self-regulation/education	"Esta campanha faz parte do compromisso da ACIBEV de implementar o Programa "Vinho com Moderação" em Portugal, cujo objetivo é promover o consumo responsável como uma norma cultural/social, de modo a prevenir e reduzir o abuso e os malefícios relacionados com o álcool. Pretende-se educar e sensibilizar jovens e adultos, ajudando-os a tomar decisões responsáveis acerca do consumo..."		https://www.acibev.pt/pt/noticias/info/acibev-lanca-campanha-vinho-com-moderacao-2018_443/ (accessed 28th May 2019)
**A52**	Associação de vinhos e espirituosas de Portugal	Discursive	Other/culture	"...ao mesmo tempo preservar o lugar cultural, ambiental e económico do vinho na sociedade portuguesa e europeia."		https://www.acibev.pt/pt/noticias/info/acibev-lanca-campanha-vinho-com-moderacao-

						2018_443/ (accessed 28th May 2019)
**A53**	Associação de vinhos e espirituosas de Portugal	Discursive	Other/war on alcohol	"As garrafas de vinho e de outras bebidas alcoólicas vão ter alertas nos rótulos para o risco de cancro. Na Europa, Portugal é uma das poucas vozes a contestar isto. Se a moda pega, o sector do vinho morre, diz a produção. Os produtores irlandeses já perceberam que têm aqui um aliado e fazem um apelo direto ao seu parceiro comunitário: "Façam ouvir a vossa voz"."		https://www.acibev.pt/pt/noticias/info/portugal-e-dos-poucos-que-contestam-alertas-de-cancro-nos-rotulos-do-vinho_444/ (accessed 28th May 2019)
**A54**	Associação de vinhos e espirituosas de Portugal	Discursive	Other/war on alcohol	"Com o Presidente do Conselho de Administração, Luís Vieira, discutiu diversos assuntos, como as ameaças anti-álcool, a fiscalidade e as barreiras ao comércio internacional."		https://www.acibev.pt/pt/noticias/info/acibev-visita-quinta-do-gradil_442/
**A55**	Associação de vinhos e espirituosas de Portugal	Discursive	Other/war on alcohol	"Mas, numa entrevista exclusiva ao Jornal Económico, George Sandeman alerta para o risco de se adotarem medidas de aumento de preço e de restrições publicitárias às bebidas alcoólicas, como tem ocorrido nos países do norte da Europa, com destaque para a Irlanda e Escócia."		https://www.acibev.pt/pt/noticias/info/presidente-da-acibev-estamos-a-assistir-a-um-fundamentalismo-antialcool_430/

						(accessed 28th May 2019)
**A56**	Associação de vinhos e espirituosas de Portugal	Discursive	Other/war on alcohol	"A ACIBEV - Associação de Vinhos e Espirituosas de Portugal - está preocupada com algumas restrições e desafios que se observam no norte da Europa."		https://www.acibev.pt/pt/noticias/info/acibev-preocupada-com-desafios-e-ameacas-europeias_429/ (accessed 28th May 2019)
**A57**	Associação de vinhos e espirituosas de Portugal	Instrumental	Relationships with leaders/health organisations	"Para celebrar os dias mais longos e quentes de Verão, a ACIBEV promoveu um novo Sunset with Wine (…) Estiveram presentes o Presidente da Comissão Parlamentar de Agricultura e Mar, os deputados Rubina Berardo, Nuno Serra, António Lima Costa, Luís Pedro Pimentel, Maurício Marques, Cristóvão Norte e Carlos Silva, o Presidente da CVR Península de Setúbal, o Inspetor-Geral da ASAE e o Subdiretor-Geral do SICAD.		https://www.acibev.pt/pt/noticias/info/acibev-preocupada-com-desafios-e-ameacas-europeias_429/ (accessed 28th May 2019)
**A58**	Associação de vinhos e espirituosas de Portugal	Discursive	Self-regulation/education	"… Na ocasião a ACIBEV reiterou a necessidade de se apostar mais na Educação, Autorregulação e fiscalização, como forma de combater o consumo nocivo do álcool, rejeitando os "Best Buys" defendidos pela Organização Mundial de Saúde."		https://www.acibev.pt/pt/noticias/info/10-anos-do-forum-nacional-alcool-e-saude_431/ (accessed 28th May 2019)

**A59**	Associação de vinhos e espirituosas de Portugal	Discursive	Self-regulation/education	" A Técnica de Relações Institucionais da ACIBEV, Márcia Azevedo Rocha, esteve em Sintra para dar formação sobre o serviço responsável de bebidas alcoólicas. (...) O objetivo primordial destas formações é minimizar os danos causados pelo consumo excessivo e/ou precoce de álcool e desenvolver uma estratégia que promova a venda responsável. Durante a formação, a Técnica abordou temas fundamentais para ajudar os profissionais a dizer não a menores ou a pessoas embriagadas, reconhecer sinais de intoxicação e criar um ambiente que incentive o consumo moderado."		https://www.acibev.pt/pt/noticias/info/formacao-de-servico-responsavel-em-sintra_428/ (accessed 28th May 2019)
**A60**	Associação de vinhos e espirituosas de Portugal	Discursive	Importance of the industry to the economy	"No seu discurso de boas-vindas, o Presidente da ACIBEV revelou a importância do setor vitivinícola para as comunidades rurais e para a economia do país. "		https://www.acibev.pt/pt/noticias/info/sunset-with-wine-vinhos-brancos_427/ (accessed 28th May 2019)
**A61**	Associação de vinhos e espirituosas de Portugal	Discursive	Other/culture	"Relembrou também que o vinho faz parte da nossa história, da nossa cultura e da nossa forma de estar na vida..."		https://www.acibev.pt/pt/noticias/info/sunset-with-wine-vinhos-brancos_427/ (accessed 28th May 2019)
**A62**	Associação de vinhos e espiritu	Discursive	Other/culture	"... e que o programa Wine in Moderation inspira ao consumo moderado e consciente de bebidas alcoólicas associado a uma dieta saudável e equilibrada e à prática de exercício físico."		https://www.acibev.pt/pt/noticias/info/sunset-with-wine-vinhos-brancos_427/

	osas de Portugal					(accessed 28th May 2019)
**A63**	Associação de vinhos e espirituosas de Portugal	Discursive	Other/war on alcohol	"Comprava uma garrafa de vinho se ela avisasse que o "álcool causa cancro" (e fosse mais cara)?"		https://www.acibev.pt/pt/noticias/info/comprava-uma-garrafa-de-vinho-se-ela-avisasse-que-o-alcool-causa-cancro-e-fosse_409/redirected to :https://observador.pt/especiais/comprava-uma-garrafa-de-vinho-se-ela-avisasse-que-o-alcool-causa-cancro-e-fosse-mais-cara/ (accessed 28th May 2019)

**A64**	Associação de vinhos e espirituosas de Portugal	Instrumental	Suppression of information	"“dizer que o vinho causa cancro é errado“, defende George Sandeman, chairman da Associação de Vinhos e Espirituosas de Portugal (ACIBEV)."		https://www.acibev.pt/pt/noticias/info/comprava-uma-garrafa-de-vinho-se-ela-avisasse-que-o-alcool-causa-cancro-e-fosse_409/redirected to :https://observador.pt/especiais/comprava-uma-garrafa-de-vinho-se-ela-avisasse-que-o-alcool-causa-cancro-e-fosse-mais-cara/ (accessed 28th May 2019)

**A65**	Associação de vinhos e espirituosas de Portugal	Discursive	Other/culture	"o setor do vinho "está em risco" porque há uma "teia de atividade, envolvendo várias entidades, que quer criar um ambiente mais restritivo para o vinho e impor uma cultura do norte sobre os países do sul da Europa". "		https://www.acibev.pt/pt/noticias/info/comprava-uma-garrafa-de-vinho-se-ela-avisasse-que-o-alcool-causa-cancro-e-fosse_409/redirected to :https://observador.pt/especiais/comprava-uma-garrafa-de-vinho-se-ela-avisasse-que-o-alcool-causa-cancro-e-fosse-mais-cara/ (accessed 28th May 2019)

**A66**	Associação de vinhos e espirituosas de Portugal	Discursive	Other/pattern of consumption	"e critica duramente qualquer política “populacional” que tenha como objetivo baixar os números do consumo total (mesmo à custa do consumo moderado) em vez de atacar os problemas relacionados com o consumo abusivo — que as associações de defesa da indústria, como a ACIBEV, dizem ser “os primeiros interessados em combater”."		https://www.acibev.pt/pt/noticias/info/comprava-uma-garrafa-de-vinho-se-ela-avisasse-que-o-alcool-causa-cancro-e-fosse_409/redirected to :https://observador.pt/especiais/comprava-uma-garrafa-de-vinho-se-ela-avisasse-que-o-alcool-causa-cancro-e-fosse-mais-cara/ (accessed 28th May 2019)

**A67**	Associação de vinhos e espirituosas de Portugal	Discursive	Not enough evidence	"Essa questão do rótulo é gravíssima porque é uma informação errada“, afirma George Sandeman, asseverando que “os estudos que existem são complexos, não há linhas conclusivas e com uma frase dessas está-se a simplificar as conclusões de uma série de estudos — o que não é informar bem o consumidor“. "		https://www.acibev.pt/pt/noticias/info/comprava-uma-garrafa-de-vinho-se-ela-avisasse-que-o-alcool-causa-cancro-e-fosse_409/redirected to :https://observador.pt/especiais/comprava-uma-garrafa-de-vinho-se-ela-avisasse-que-o-alcool-causa-cancro-e-fosse-mais-cara/ (accessed 28th May 2019)

**A68**	Associação de vinhos e espirituosas de Portugal	Discursive	Other/culture	"Nos países do norte da Europa há uma cultura de consumo de bebidas e de vinho que, de um modo geral, é caracterizada por muito consumo em poucos dias. Em Portugal, o vinho faz parte da mesa, faz parte da gastronomia, faz parte da dieta, com moderação — faz parte da nossa cultura, desde a produção até ao consumo."		https://www.acibev.pt/pt/noticias/info/comprava-uma-garrafa-de-vinho-se-ela-avisasse-que-o-alcool-causa-cancro-e-fosse_409/redirected to :https://observador.pt/especiais/comprava-uma-garrafa-de-vinho-se-ela-avisasse-que-o-alcool-causa-cancro-e-fosse-mais-cara/ (accessed 28th May 2019)

**A69**	Associação de vinhos e espirituosas de Portugal	Discursive	Self-regulation/education	" ...mas as associações do setor contrapõem com a auto-regulação e com investimento em campanhas como o Wine in Moderation (vinho com moderação) e outras iniciativas, como ações de formação a funcionários de bares e restaurantes sobre como a melhor forma de, por exemplo, recusar servir bebidas alcoólicas a quem já bebeu demasiado..."		https://www.acibev.pt/pt/noticias/info/comprava-uma-garrafa-de-vinho-se-ela-avisasse-que-o-alcool-causa-cancro-e-fosse_409/redirected to :https://observador.pt/especiais/comprava-uma-garrafa-de-vinho-se-ela-avisasse-que-o-alcool-causa-cancro-e-fosse-mais-cara/ (accessed 28th May 2019)

**A70**	Associação de vinhos e espirituosas de Portugal	Discursive	Other/pattern of consumption	"“A própria OMS reconheceu que países como Portugal têm padrões de consumo mais seguros. Efetivamente consumimos muito mas temos um padrão de consumo de baixo risco."		https://www.acibev.pt/pt/noticias/info/comprava-uma-garrafa-de-vinho-se-ela-avisasse-que-o-alcool-causa-cancro-e-fosse_409/redirected to :https://observador.pt/especiais/comprava-uma-garrafa-de-vinho-se-ela-avisasse-que-o-alcool-causa-cancro-e-fosse-mais-cara/ (accessed 28th May 2019)

**A71**	Associação de vinhos e espirituosas de Portugal	Discursive	Other/pattern of consumption	"...George Sandeman, relembrando que "não são restrições para acabar com o consumo abusivo, mas apenas medidas para reduzir o consumo".		https://www.acibev.pt/pt/noticias/info/comprava-uma-garrafa-de-vinho-se-ela-avisasse-que-o-alcool-causa-cancro-e-fosse_409/redirected to :https://observador.pt/especiais/comprava-uma-garrafa-de-vinho-se-ela-avisasse-que-o-alcool-causa-cancro-e-fosse-mais-cara/ (accessed 28th May 2019)

**A72**	Associação de vinhos e espirituosas de Portugal	Discursive	Unfair to drinkers	"Medidas como o preço mínimo vão penalizar os consumidores moderados, diz a responsável,..."		https://www.acibev.pt/pt/noticias/info/comprava-uma-garrafa-de-vinho-se-ela-avisasse-que-o-alcool-causa-cancro-e-fosse_409/redirected to :https://observador.pt/especiais/comprava-uma-garrafa-de-vinho-se-ela-avisasse-que-o-alcool-causa-cancro-e-fosse-mais-cara/ (accessed 28th May 2019)

**A73**	Associação de vinhos e espirituosas de Portugal	Discursive	Other/pattern of consumption	""O foco não pode ser acabar com o consumo. Estas medidas vão abarcar a população em geral. Provavelmente o consumo per capita vai cair, tal como as nossas exportações, mas não vamos resolver o problema do consumo problemático: uma pessoa que tem um problema de adição, em última análise, bebe álcool das feridas ou perfumes, ou destila em casa -- como acontece nos países onde já foram aplicadas medidas deste género" Ana Isabel Alves, secretária-geral da ACIBEV "		https://www.acibev.pt/pt/noticias/info/comprava-uma-garrafa-de-vinho-se-ela-avisasse-que-o-alcool-causa-cancro-e-fosse_409/redirected to :https://observador.pt/especiais/comprava-uma-garrafa-de-vinho-se-ela-avisasse-que-o-alcool-causa-cancro-e-fosse-mais-cara/ (accessed 28th May 2019)

**A74**	Associação de vinhos e espirituosas de Portugal	Discursive	Other/culture	A ACIBEV, que representa mais de 80 empresas e 600 milhões de euros em faturação, garante que está a fazer mais do que "defender um setor económico", está a "defender a nossa cultura".		https://www.acibev.pt/pt/noticias/info/comprava-uma-garrafa-de-vinho-se-ela-avisasse-que-o-alcool-causa-cancro-e-fosse_409/redirected to :https://observador.pt/especiais/comprava-uma-garrafa-de-vinho-se-ela-avisasse-que-o-alcool-causa-cancro-e-fosse-mais-cara/ (accessed 28th May 2019)

**A75**	Associação de vinhos e espirituosas de Portugal	Discursive	Other/culture	" Ana Isabel Alves diz que “o objetivo final dos lóbis anti-álcool é privar-nos de beber um copo de vinho à refeição”. "		https://www.acibev.pt/pt/noticias/info/comprava-uma-garrafa-de-vinho-se-ela-avisasse-que-o-alcool-causa-cancro-e-fosse_409/redirected to :https://observador.pt/especiais/comprava-uma-garrafa-de-vinho-se-ela-avisasse-que-o-alcool-causa-cancro-e-fosse-mais-cara/ (accessed 28th May 2019)

**A76**	Associação de vinhos e espirituosas de Portugal	Discursive	Other/culture	"“Não podemos embarcar nestas ondas, os países do sul têm de se unir, porque quando as medidas forem aplicadas não vão fazer distinção entre o vinho e o vodka, vai tudo a eito”, alerta a responsável."		https://www.acibev.pt/pt/noticias/info/comprava-uma-garrafa-de-vinho-se-ela-avisasse-que-o-alcool-causa-cancro-e-fosse_409/redirected to :https://observador.pt/especiais/comprava-uma-garrafa-de-vinho-se-ela-avisasse-que-o-alcool-causa-cancro-e-fosse-mais-cara/ (accessed 28th May 2019)

**A77**	Associação de vinhos e espirituosas de Portugal	Instrumental	Internal constituency recruitment	"A Secretária-Geral da ACIBEV, Ana Isabel Alves, esteve em Bruxelas para participar nas reuniões dos grupos de trabalho do Comité Vins, a Federação Europeia dos Vinhos. Em discussão estiveram temas como o Programa de Promoção em Países Terceiros, a reforma da PAC, a revisão de diversos Regulamentos do Setor dos vinhos e bebidas aromatizadas, as ameaças anti álcool, as negociações de Acordos de Comércio Livre e questões fiscais."		https://www.acibev.pt/pt/noticias/info/acibev-em-bruxelas-para-as-reunioes-dos-grupos-de-trabalho-do-ceev_411/ (accessed 28th May 2019)
**A78**	Associação de vinhos e espirituosas de Portugal	Instrumental	Relationships with leaders/health organisations	"O novo Secretário-Geral do SICAD, Manuel Cardoso, convidou ainda a Secretária-Geral da ACIBEV a fazer um ponto de situação sobre a proposta de autorregulação do setor de bebidas alcoólicas em matéria de ingredientes e informação nutricional."		https://www.acibev.pt/pt/noticias/info/acibev-presente-no-plenario-anual-do-forum-nacional-alcool-e-saude_403/ (accessed 28th May 2019)
**A79**	Associação de vinhos e espirituosas de Portugal	Discursive	Self-regulation/education	"Queixa-se, por exemplo, da tendência de taxar demasiado as bebidas alcoólicas para desincentivar o consumo por oposição a educar. Sublinha que apoia a decisão de ser proibido consumir bebidas alcoólicas com menos de 18 anos, "mesmo porque há evidências científicas que demonstram os seus efeitos", mas acredita que não vem mal ao mundo - pelo contrário - se ainda antes desta idade e em ambiente controlado os pais forem educando, ensinando que se pode beber um copo de vinho de vez em quando"		https://www.acibev.pt/pt/noticias/info/e-o-vinho-que-leva-os-pontapes-quando-a-europa-decide-fazer-alguma-coisa_378/red

						irected to: https://www.dn.pt/portugal/interior/e-o-vinho-que-leva-os-pontapes-quando-a-europa-decide-fazer-alguma-coisa-9258525.html (accessed 28th May 2019)
**A80**	Associação de vinhos e espirituosas de Portugal	Discursive	Self-regulation/education	"...a Secretária-Geral da ACIBEV fez referência à importância da educação como medida de prevenção do consumo nocivo entre os jovens"		https://www.acibev.pt/pt/noticias/info/sunset-with-wine_377/ (accessed 28th May 2019)
**A81**	Associação de vinhos e espirituosas de Portugal	Discursive	Self-regulation/education	"A ACIBEV esteve presente na Essência do Vinho 2018 com o seu programa de responsabilidade social Wine in Moderation. Apresentou, também, aos profissionais que visitaram o evento, o seu programa de serviço responsável de bebidas alcoólicas “O Serviço Responsável faz o Negócio Sustentável”."		https://www.acibev.pt/pt/noticias/info/essencia-do-vinho-palacio-da-bolsa-porto-2018_371/ (accessed 28th May 2019)

**A82**	Associação de vinhos e espirituosas de Portugal	Instrumental	Relationships with leaders/health organisations	"...o Presidente George Sandeman e a Secretária-Geral da ACIBEV, Ana Isabel Alves, reuniram com a Secretária de Estado dos Assuntos Europeus, Ana Paula Zacarias, com quem abordaram a política europeia para o álcool. "		https://www.acibev.pt/pt/noticias/info/essencia-do-vinho-palacio-da-bolsa-porto-2018_371/ (accessed 28th May 2019)
**A83**	Associação de vinhos e espirituosas de Portugal	Instrumental	Relationships with leaders/health organisations	"A Secretária-Geral da ACIBEV, Ana Isabel Alves, esteve presente na Sessão de Apresentação do Relatório Anual sobre a Situação do País em Matéria de Drogas e Toxicodependência e do Relatório Anual sobre a Situação do País em Matéria de Álcool 2016, que decorreu no dia 7 de fevereiro de 2018, na Assembleia da República."		https://www.acibev.pt/pt/noticias/info/sicad-apresentacao-do-relatorio-anual-sobre-a-situacao-do-pais-em-materia-de-al_366/ (accessed 28th May 2019)
**A84**	Associação de vinhos e espirituosas de Portugal	Instrumental	Internal constituency recruitment	"...a Comissão Álcool e Sociedade do Comité Vins, presidida por George Sandeman, Presidente da ACIBEV. Em destaque estiveram várias iniciativas que afetam negativamente o setor das bebidas alcoólicas, como os relatórios da OCDE, os trabalhos da OMS e a nova lei da Irlanda."		https://www.acibev.pt/pt/noticias/info/reuniao-comissao-alcool-e-sociedade-do-comite-vins_346/ (accessed 28th May 2019)

**A85**	Associação de vinhos e espirituosas de Portugal	Instrumental	Relationships with leaders/health organisations	" O Presidente e a Secretária-Geral da ACIBEV reuniram, no dia 19 de Janeiro, em Paris, com o Embaixador de Portugal junto da OCDE. George Sandeman e Ana Isabel Alves transmitiram as preocupações do setor do Vinho e das Bebidas Espirituosas..."		https://www.acibev.pt/pt/noticias/noticias_68/7/ (accessed 28th May 2019)
**A86**	Associação de vinhos e espirituosas de Portugal	Discursive	Self-regulation/education	"O programa trabalha para promover o consumo responsável como uma norma cultural/social, de modo a prevenir e reduzir o abuso e os malefícios relacionados com o álcool. O nosso objetivo é educar jovens e adultos, ajudando-os a tomar decisões responsáveis acerca do consumo, ao mesmo tempo que queremos preservar o lugar cultural, ambiental e económico do vinho na sociedade europeia."		https://www.acibev.pt/pt/responsabilidade_social/wine-in-moderation_88/ (accessed 28th May 2019)
**A87**	Associação de vinhos e espirituosas de Portugal	Instrumental	Amplification of information	"O consumo moderado aumenta as lipoproteínas de elevada densidade (HDL – bom colesterol), que se ligam ao colesterol e o encaminham para o fígado, para eliminação ou reprocessamento - evitam a deposição de colesterol nas artérias e a formação de placa de ateroma (...)Além disso, o álcool tem efeito geral anti-inflamatório que afeta os vasos sanguíneos de forma positiva, retardando assim o aparecimento de aterosclerose.O vinho é a bebida alcoólica que apresenta maior concentração de compostos biologicamente ativos, particularmente compostos fenólicos (resveratrol, quercetina, epicatequina, taninos, antocianinas...), que são responsáveis por características organoléticas, como sabor, adstringência e corpo. Estes compostos têm ação antioxidante e parecem inibir a oxidação das LDL, prevenindo o desenvolvimento de aterosclerose, a principal causa de doença coronária. Além disso, os compostos fenólicos combatem a ação dos radicais livres, que estão na		https://www.acibev.pt/conteudos/responsabilidade_social/info.aspx?id=110&title=alcool-e-saude&idioma=pt (accessed 28th May 2019)

				base do envelhecimento precoce e do desenvolvimento de patologias como Parkinson, Alzheimer e alguns tipos de cancro."		
**A88**	Associação de vinhos e espirituosas de Portugal	Instrumental	Amplification of information	"As evidências científicas reunidas até à data indicam que o consumo moderado de álcool (1 a 2 bebidas/dia) está associado a uma diminuição do risco (cerca de 30%) de diabetes tipo 2, comparativamente aos abstémios ou indivíduos com baixo consumo."		https://www.acibev.pt/conteudos/responsabilidade_social/info.aspx?id=110&title=alcool-e-saude&idioma=pt (accessed 28th May 2019)
**A89**	Associação de vinhos e espirituosas de Portugal	Discursive	Self-regulation/education	"O Programa VCM baseia-se em factos científicos,na educação e na autorregulação para organizar e capacitar toda a cadeia internacional de valor do vinho, sensibilizando e reforçando os conhecimentos sobre os padrões de consumo de álcool responsável e o consumo moderado de vinho."		https://www.acibev.pt/multimedia/1/documentos/117/19410203599361G.pdf?title=download&idioma=pt

						(accessed 28th May 2019)
**A90**	Associação de vinhos e espirituosas de Portugal	Discursive	Self-regulation/education	"Com o intuito de reduzir os malefícios do consumo abusivo de bebidas alcoólicas, a ACIBEV está presente em diversos eventos com o projeto WIM by ACIBEV – testes de alcoolemia, cujo objetivo é sensibilizar sobre o consumo de bebidas alcoólicas e a condução."		https://www.acibev.pt/conteudos/responsabilidade_social/info.aspx?id=267&title=testes-de-alcoolemia&idioma=pt (accessed 28th May 2019)
**A91**	Associação de vinhos e espirituosas de Portugal	Instrumental	Community involvement	" A campanha de responsabilidade social da ACIBEV, lançada em 2017, apela ao consumo responsável e moderado de bebidas alcoólicas. Em 2018, o filme estará em exibição nos Cinemas NOS e na SIC e SIC Notícias, entre o dia 11 de outubro e o dia 5 de dezembro."		https://www.acibev.pt/pt/noticias/info/acibev-lanca-novo-filme-de-responsabilidade-social_341/ (accessed 28th May 2019)
**A92**	Associação dos cervejeiros de Portugal	Discursive	Self-regulation/education	"Seja reponsável. Beba com moderação"		https://www.cervejeirosdeportugal.pt/ (accessed 28th May 2019)

**A93**	Associação dos cervejeiros de Portugal	Instrumental	Internal constituency recruitment	"A The Brewers of Europe é uma organização que reúne associações nacionais de 29 países europeus, sendo a Cervejeiros de Portugal uma destas associadas, e representa os interesses de 9.000 empresas cervejeiras existentes na Europa."		https://www.cervejeirosdeportugal.pt/2edicao-forum-the-brewers-of-europe/ (accessed 28th May 2019)
**A94**	Associação dos cervejeiros de Portugal	Instrumental	Relationships with leaders/health organisations	"Cervejeiros de Portugal é associado da FIPA-Federação das Indústrias Portuguesas Agroalimentares desde o dia 1 de janeiro de 2005 presidindo desde 2010 ao seu Conselho Fiscal. É ainda sócia fundadora (acionista) da EMBOPAR-Embalagens de Portugal, SGPS, S.A. desde 1996, tendo participado na constituição da Sociedade Ponto Verde (SPV), entidade responsável pela gestão do Sistema Integrado de Gestão de Resíduos de Embalagens (SIGRE"		https://www.cervejeirosdeportugal.pt/representacoes/ (accessed 28th May 2019)
**A95**	Associação dos cervejeiros de Portugal	Instrumental	Relationships with leaders/health organisations	"Cervejeiros de Portugal mantém contacto privilegiado (parceria) com o Ministério da Saúde (IDT _ Instituto da Droga e da Toxicodependência) no âmbito do Fórum Nacional Álcool & Saúde - SICAD, onde pertence à sua Comissão Executiva e é membro do Conselho Nacional da Coordenação Nacional para os Problemas da Droga, da Toxicodependências e do Uso Nocivo do Álcool"		https://www.cervejeirosdeportugal.pt/representacoes/ (accessed 28th May 2019)
**A96**	Associação dos cervejeiros de Portugal	Instrumental	Community involvement	"Possui ainda parcerias ativas com a CONFAP _ Confederação Nacional Das Associações de Pais, ANP – Associação Nacional de Professores e Instituto Português da Juventude - IPDJ no âmbito de programas educacionais para pais relativo ao consumo nocivo de álcool e com a AHRESP e APED no âmbito do seu Código de Autorregulação para a Comunicação Comercial."		https://www.cervejeirosdeportugal.pt/representacoes/ (accessed 28th May 2019)

**A97**	Associação dos cervejeiros de Portugal	Instrumental	Amplification of information	"A cerveja é realmente saudável? A palavra chave é: moderação. Como em tudo na vida, existem malefícios associados ao consumo excessivo de cerveja. Os estudos mostram que o consumo de um a três copos de cerveja por dia, aliados a um estilo de vida saudável, pode ajudar a reduzir o riscos de ataques cardíacos, de AVCs, diabetes, Alzheimer, pedras nos rins e ainda cataratas!"		https://www.cervejeirosdeportugal.pt/factosecuriosidades/ (accessed 28th May 2019)
**A98**	Associação dos cervejeiros de Portugal	Instrumental	Amplification of information	"A cerveja faz “barriga”?Novamente, a moderação. Não existe uma correlação direta entre o aumento de peso e o consumo moderado de cerveja. O que existe, é uma relação entre um estilo de vida pouco saudável e o aumento de peso. A Cerveja tem calorias, pelo que o seu consumo em excesso pode levar a um aumento de peso. No entanto, o teor calórico da Cerveja varia muitíssimo (tanto quantas as cervejas existentes!) e pode ir desde as 30kcal por cada 100ml até às 200kcal. Sabias que a mesma quantidade de leite meio-gordo tem, em média, 50kcal? Talvez a “barriga de cerveja” tenha mais a ver com as batatas fritas, pipocas, amendoins e snacks variados com que se acompanham as cervejas… Não?"		https://www.cervejeirosdeportugal.pt/factosecuriosidades/ (accessed 28th May 2019)
**A99**	Associação dos cervejeiros de Portugal	Instrumental	Amplification of information	"Cerveja depois do exercício ajuda a reduzir a dor? Sim! Beber um a dois copos de cerveja tem um efeito benéfico sobre a dor pós-exercício físico. A quantidade de água existente na cerveja (cerca de 90%) ajuda a compensar a desidratação. E a presença de sais minerais e outros nutrientes abundantes na cerveja restabelecem o equilíbrio ácido-base. Incrível, não é?"		https://www.cervejeirosdeportugal.pt/factosecuriosidades/ (accessed 28th May 2019)

**A100**	Associação dos cervejeiros de Portugal	Instrumental	Amplification of information	"A cerveja faz bem à pele. De acordo com os escritos de Plínio, de há 2000 anos atrás, as mulheres do Antigo Egipto utilizavam a espuma da cerveja para tornar a pele mais fresca e clara. Apesar de insólito, este uso cosmético da cerveja tem um fundamento científico: a cerveja contém ácido fosfórico que é um componente essencial no crescimento dos tecidos celulares e, assim, contribui para a manutenção de uma pele saudável"		https://www.cervejeirosdeportugal.pt/factosecuriosidades/ (accessed 28th May 2019)
**A101**	Associação dos cervejeiros de Portugal	Instrumental	Amplification of information	Cerveja e saúde "A cerveja é uma bebida saudável e que faz parte da dieta do Homem desde tempos ancestrais"		https://www.cervejeirosdeportugal.pt/beneficios/ (accessed 28th May 2019)
**A102**	Associação dos cervejeiros de Portugal	Discursive	Self-regulation/education	"...é hoje bem nítido que elas não são apenas boas para beber, como também fazem bem à nossa saúde, desde que consumidas moderada e regularmente, isto é, não mais de 2 garrafas de 33cl por dia."		https://www.cervejeirosdeportugal.pt/beneficios/ (accessed 28th May 2019)
**A103**	Associação dos cervejeiros de Portugal	Instrumental	Amplification of information	"A cerveja como elemento de uma dieta saudável...Por outro lado, a cerveja pode contribuir positivamente para uma dieta saudável..."		https://www.cervejeirosdeportugal.pt/beneficios/ (accessed 28th May 2019)
**A104**	Associação dos cervejeiros de Portugal	Instrumental	Amplification of information	"Para além destes ingredientes, a cerveja é basicamente água (cerca de 93%), constituindo pois uma fonte excepcional deste bem essencial à vida, servindo igualmente para saciar a sede. Como já atrás ficou dito, a cerveja pode integrar uma dieta equilibrada, fornecendo vitaminas essenciais e diversos sais minerais, podendo realçar-se o seu elevado teor de potássio e		https://www.cervejeirosdeportugal.pt/beneficios/ (accessed 28th May 2019)

				baixo valor de sódio, indispensável a uma tensão sanguínea normalizada"		
**A105**	Associação dos cervejeiros de Portugal	Instrumental	Amplification of information	""Saciar" a sede com cerveja<br>A água é um componente essencial do sangue, linfa, secreções corporais e líquido intracelular. Constitui cerca de 60% do peso do corpo humano adulto, sendo aproximadamente 2/3 dessa quantidade água intracelular. Pelo facto de conter mais de 90% de água, a cerveja pode contribuir para as necessidades diárias de água do corpo humano, que são de 1,5 a 2,0 litros."		https://www.cervejeirosdeportugal.pt/beneficios/ (accessed 28th May 2019)
**A106**	Associação dos cervejeiros de Portugal	Instrumental	Self-regulation/education	"MARKETING E AUTO REGULAÇÃO ::<br><br>Alargamento da monitorização sistemática da comunicação comercial dos cervejeiros portugueses<br><br>1. Os Cervejeiros de Portugal possuem, desde 2007, um acordo de monitorização sistemática pelo ICAP de toda a comunicação comercial dos cervejeiros portugueses em TV no âmbito do seu Código de Auto-Regulação dos Cervejeiros Portugueses para a Comunicação Comercial (Protocolo ICAP/Cervejeiros de Portugal assinado em 29 de Maio de 2007)."		https://www.cervejeirosdeportugal.pt/marketing-e-auto-regulacao/ (accessed 28th May 2019)

**A107**	Associação dos cervejeiros de Portugal	Discursive	Self-regulation/education	"O consumo de bebidas alcoólicas, e em particular de cerveja, não é intrinsecamente um problema. O problema surge do seu consumo abusivo e que varia consideravelmente de país para país. Grande parte das pessoas bebe cerveja ou bebidas alcoólicas de uma forma moderada e responsável, havendo, no entanto, uma minoria que as consome irresponsavelmente, possivelmente pelas razões erradas ou simplesmente fazendo-o de forma excessiva e com frequência"		https://www.cervejeirosdeportugal.pt/consumo-e-responsavel/ (accessed 28th May 2019)
**A108**	Associação dos cervejeiros de Portugal	Discursive	Discriminatory among the producers	"Existe em Portugal, tal como na maioria dos países europeus, legislação que refere uma idade mínima legal de consumo de bebidas alcoólicas (DL nº 106/2015) e que no caso nacional é de 18 anos. De notar, que a nossa legislação não distingue essa idade mínima legal de acordo com o tipo de bebidas alcoólicas ou com o seu grau alcoólico. Em Itália por ex., a idade mínima de consumo de cerveja é de 16 anos e a de consumo de bebidas espirituosas (maior teor em álcool) de 18 anos. O mesmo se passa na Bélgica e na Alemanha."		https://www.cervejeirosdeportugal.pt/cerveja-jovens/ (accessed 28th May 2019)
**A109**	Associação dos cervejeiros de Portugal	Discursive	Self-regulation/education	" é absolutamente fundamental apostar em medidas indutoras de alterações comportamentais através da formação e educação de todos os intervenientes (jovens, donos de estabelecimentos, associações de barmen, indústria da noite, etc) e que aposte em programas educacionais dirigidos aos menores e às famílias, a começar pelo próprio meio escolar, que permita combater o consumo ilegal de bebidas alcoólicas. De facto, estudos de opinião revelam que em Portugal, é cada vez mais precoce o consumo de bebidas alcoólicas por menores de idade. Urge atuar através da educação/sensibilização bem como de uma fiscalização mais eficaz da legislação existente"		https://www.cervejeirosdeportugal.pt/cerveja-jovens/ (accessed 28th May 2019)

**A110**	Associação dos cervejeiros de Portugal	Instrumental	Community involvement	"A Cervejeiros de Portugal está atenta a este problema e desenvolve atividades, em parceria com stakeholders da sociedade civil, delineando e financiando programas e eventos que procuram atingir uma relação causa-efeito que contribua para uma resposta eficaz no combate ao consumo abusivo de bebidas alcoólicas por jovens."		https://www.cervejeirosdeportugal.pt/cerveja-jovens/ (accessed 28th May 2019)
**A111**	Associação dos cervejeiros de Portugal	Instrumental	Community involvement	"A Cervejeiros de Portugal e os parceiros CONFAP, ANP e IPDJ, desenvolvem em conjunto o projeto FALAR CLARO, que tem como propósito auxiliar professores, educadores e outros técnicos com ferramentas para abordar o consumo responsável de álcool e os perigos para a saúde do consumo de álcool pelos jovens."		https://www.cervejeirosdeportugal.pt/cerveja-jovens/ (accessed 28th May 2019)
**A112**	Associação dos cervejeiros de Portugal	Instrumental	Amplification of information	"As iniciativas levadas a cabo pelos produtores de cerveja em parceria com outras organizações têm contribuído para um aumento da consciencialização pública para o perigo de conduzir sob efeito de álcool, que se têm traduzido numa tendência de decréscimo do factor álcool relacionado com acidentes e fatalidades, segundo os resultados do Worldwide Brewing Alliance Drinking and Driving Report. "		https://www.cervejeirosdeportugal.pt/cerveja-e-conducao/ (accessed 28th May 2019)
**A113**	Associação dos cervejeiros de Portugal	Instrumental	Relationships with leaders/health organisations	"Os Cervejeiros de Portugal vão dinamizar um simpósio satélite no XVIII Congresso de Nutrição e Alimentação, promovido pela Associação Portuguesa de Nutrição, nos dias 16 e 17 de maio, no Centro de Congressos da Alfândega do Porto, sob o mote "O Valor da Nutrição."		https://www.cervejeirosdeportugal.pt/cervejeiros-de-portugal-debatem-cerveja-dieta-mediterranica-no-xviii-congresso-de-nutricao-e-alimentacao/

						(accessed 28th May 2019)
**A114**	Associação dos cervejeiros de Portugal	Instrumental	Credibility of information	"“Cerveja & Dieta Mediterrânica” será o tema a abordar neste simpósio satélite, que contará com um debate dedicado à questão “Cerveja: o que a evidência científica tem demonstrado?”, com a presença da nutricionista e Professora Associada com Agregação da NOVA Medical School - Faculdade de Ciências Médicas, Conceição Calhau, e da Professora Associada na Escola Superior de Biotecnologia do Porto e Diretora do Centro de Investigação CBQF - Centro de Biotecnologia e Química Fina, Manuela Pintado."		https://www.cervejeirosdeportugal.pt/cervejeiros-de-portugal-debatem-cerveja-dieta-mediterranica-no-xviii-congresso-de-nutricao-e-alimentacao/ (accessed 28th May 2019)
**A115**	Associação dos cervejeiros de Portugal	Instrumental	Amplification of information	"Ainda que visto com algum ceticismo, o consumo de cerveja é benéfico para a saúde, desde que moderado. É uma bebida fermentada através de um processo natural que é rica em diferentes vitaminas, minerais e composto antioxidantes		https://www.cervejeirosdeportugal.pt/consumoresponsavel-3/ (accessed 28th May 2019)

**A116**	Associação dos cervejeiros de Portugal	Instrumental	Amplification of information	Está cientificamente provado que o consumo moderado de bebidas com baixo teor alcoólico é benéfico para a prevenção de doenças cardiovasculares - que, infelizmente, ainda vão sendo das primeiras causas de morte entre os portugueses. E, para sublinhar ainda mais vantagens, o aumento dos valores de HDL (bom colesterol), redução do processo é algo que podemos encontrar no consumo moderado de cerveja.		https://www.cervejeirosdeportugal.pt/consumoresponsavel-3/ (accessed 28th May 2019)
**A117**	Associação dos cervejeiros de Portugal	Instrumental	Amplification of information	"A presença de antioxidantes na bebida, os chamados polifenóis, que provêm não só do malte, como do lúpulo têm sido comprovados como eficazes por estudos no aumento da sensibilidade à insulina, podendo ser útil para os diabéticos Tipo 2, melhorando os seus perfis glicémicos. Além disso, um dos minerais mais importantes na cerveja é o silício. Este oligoelemento desempenha um papel importantíssimo na densidade mineral óssea, como na prevenção de fracturas e manutenção de colagénio, encontrando-se abundantemente no seu malte. A ingestão deste mineral traz benefícios acrescidos às mulheres pós-menopausa, onde habitualmente surge a osteoporose."		https://www.cervejeirosdeportugal.pt/consumoresponsavel-3/ (accessed 28th May 2019)
**A118**	Associação dos cervejeiros de Portugal	Instrumental	Amplification of information	"Há vários estudos que mostram a eficácia do seu consumo antes (devido à presença de hidratos de carbono assegurando as reservas de glicogénio muscular), durante (prevenção da fadiga) e no final (como recuperador e preventivo dos processos inflamatórios recorrentes da prática do exercício físico de maior intensidade)."		https://www.cervejeirosdeportugal.pt/consumoresponsavel-3/ (accessed 28th May 2019)

**A119**	Associação dos cervejeiros de Portugal	Discursive	Government is unreasonable	"A fiscalidade dentro do setor atingiu o pico máximo, é incomportável que possa continuar a aumentar. O setor não aceita menos do que o congelamento do IABA [imposto sobre o álcool e as bebidas alcoólicas] no Orçamento do Estado para o próximo ano"		https://www.cervejeirosdeportugal.pt/congelamento-do-imposto-aplicada-a-cerveja-no-oe-2019/ (accessed th May 2019)
**A120**	Associação dos cervejeiros de Portugal	Discursive	Discriminatory among the producers	"E, claro, há ainda a questão da justiça fiscal em relação ao vinho, cujo IEC se mantém em zero."		https://www.cervejeirosdeportugal.pt/congelamento-do-imposto-aplicada-a-cerveja-no-oe-2019/ (accessed th May 2019)
**A121**	Associação dos cervejeiros de Portugal	Discursive	Importance of the industry to the economy	"Francisco Gírio lembra que a cerveja assegura mais de 82 mil postos de trabalho, diretos e indiretos, além de ser fundamental para a produção agrícola nacional: no ano passado, o setor comprou quase 43 mil toneladas de cevada em Portugal e mais de seis toneladas de lúpulo. E só não compra mais porque não há."		https://www.cervejeirosdeportugal.pt/congelamento-do-imposto-aplicada-a-cerveja-no-oe-2019/ (accessed th May 2019)

**A122**	Associação dos cervejeiros de Portugal	Discursive	Illicit trade	"“Nunca um governo espanhol aumentou os impostos especiais sobre o consumo à cerveja! E esta começa a ser uma diferença enorme quando estamos a concorrer nos mercados externos”, defende Francisco Gírio, que lamenta que o setor cervejeiro “seja o único, da fileira de valor nacional, com um aumento contínuo de impostos”, apesar de contribuir com mais de mil milhões de euros anuais para a economia nacional."		https://www.cervejeirosdeportugal.pt/congelamento-do-imposto-aplicada-a-cerveja-no-oe-2019/ (accessed th May 2019)
**A123**	Associação dos cervejeiros de Portugal	Instrumental	Amplification of information	"Nutrientes e seus contributos para a saúde: Álcool: Pode prevenir o aparecimento de diabetes do tipo 2 por reduzir os níveis de insulina. Proteção do organismo contra a doença coronária, desde que ingerido em quantidades moderadas (estudos mostram que a incidência pode ser menor em consumidores moderados do que em abstémios)."		https://www.cervejeirosdeportugal.pt/consumoresponsavel-3/ (accessed 28th May 2019)
**A124**	Associação dos cervejeiros de Portugal	Instrumental	Amplification of information	"Piridoxina e folatos: Diminuição da incidência de doenças coronárias. Polifenóis: Capacidade antioxidante, importante na prevenção de doenças cardio-vasculares. Possível papel quimiopreventivo. Ácido fólico e outras Vitaminas do complexo B: Equilíbrio da saúde do sistema nervoso, designadamente prevenção de acidentes cardio-vasculares. Metabolismo dos glúcidos. Prevenção da anemia. Reforço imunológico. Digestão. Fibra solúvel: Regulação dos níveis de colesterol e de glicose no sangue. Desenvolvimento da flora intestinal. Diminuição da incidência do cancro do cólon. (A cerveja tem tanta fibra solúvel quanto um sumo de laranja). Silício: Maior densidade mineral óssea. Prevenção da osteoporose. Potássio e sódio: Regulação da tensão arterial, por estarem presentes na cerveja em quantidades proporcionais. Magnésio: Metabolismo dos		https://www.cervejeirosdeportugal.pt/consumoresponsavel-3/ (accessed 28th May 2019)

				músculos, (uma dose de 33 cl de cerveja fornece cerca de 8% da dose diária recomendada de magnésio). "		
**A125**	Associação dos cervejeiros de Portugal	Discursive	Self-regulation/education	"Apesar de todas as qualidades desta bebida, nunca é demais alertar para os perigos de um consumo abusivo de álcool, pelo que a responsabilidade e moderação são tónicas indispensáveis quando se fala em beber cerveja."		https://www.cervejeirosdeportugal.pt/a-cerveja-engorda/ (accessed 28th May 2019)
**A126**	Associação dos cervejeiros de Portugal	Instrumental	Amplification of information	"Numa altura em que a alimentação ocidental é pobre na maioria dos constituintes da cerveja e excessivamente rica em lípidos, açucares e sódio, que existem em pequenas quantidades na cerveja, esta bebida pode, se consumida de forma moderada e às refeições, ser um óptimo complemento numa dieta saudável."		https://www.cervejeirosdeportugal.pt/a-cerveja-engorda/ (accessed 28th May 2019)

**A127**	Associação dos cervejeiros de Portugal	Instrumental	Amplification of information	"Evidências científicas: No estudo científico da University College London, que observou cerca de 2000 homens e mulheres, não foi notada uma relação entre o consumo moderado de cerveja e o aumento significativo da linha de cintura ou do volume de corpo em geral, ou seja, constatou que a cerveja, consumida em doses moderadas, não é factor de obesidade. "		https://www.cervejeirosdeportugal.pt/a-cerveja-engorda/ (accessed 28th May 2019)
**A128**	Associação dos cervejeiros de Portugal	Instrumental	Credibility of information	"Mais: de acordo com os dois professores da Faculdade de Ciências da Nutrição e Alimentação da Universidade do Porto, se compararmos com outras bebidas alcoólicas, a cerveja é a melhor opção: não tem açúcar, tem um baixo grau de álcool, tem mais de 90% de água, é uma fonte de vitaminas do grupo B e de alguns minerais, e tem efeito diurético."		https://www.cervejeirosdeportugal.pt/a-cerveja-engorda/ (accessed 28th May 2019)
**A129**	Organização Interprofissional do Vinho de Portugal	Discursive	Self-regulation/education	"O consumo Moderado e Responsável sendo SIGNATÁRIA DO PROGRAMA "WINE IN MODERATION"		https://www.viniportugal.pt/ResponsabilidadeSocial (accessed 30th May 2019)
**A130**	Organização Interprofissional do Vinho de Portugal	Discursive	Self-regulation/education	"Pretende educar os "stakeholders" e o público em geral sobre os riscos sociais e de saúde do consumo excessivo e do uso impróprio de álcool, ao mesmo tempo que encoraja uma mudança cultural na abordagem ao consumo, fazendo da moderação uma moda. "		https://www.viniportugal.pt/ResponsabilidadeSocial (accessed 30th May 2019)

**A131**	Organização Interprofissional do Vinho de Portugal	Discursive	Self-regulation/education	"A ViniPortugal é responsável pela gestão e comunicação de duas marcas (…)"a Copo!", que incentiva o consumo Responsável e Moderado de vinho a copo. "		https://www.viniportugal.pt/PressKitPt (accessed 30th May 2019)
**A132**	SICAD	Instrumental	Relationships with leaders/health organisations	"Pretende-se manter, a nível nacional, uma plataforma representativa de todas as partes interessadas da sociedade civil que se comprometam a reforçar as ações necessárias à minimização dos danos provocados pelo consumo nocivo de álcool, nomeadamente nas áreas referidas no Plano Nacional para a Redução dos Comportamentos Aditivos e das Dependências."		http://www.sicad.pt/PT/FNAS/Paginas/default.aspx (accessed 4th June 2019)
**A133**	SICAD	Instrumental	Relationships with leaders/health organisations	"O FNAS pretende assegurar uma estreita colaboração com todos os intervenientes que se comprometeram a desenvolver um conjunto de ações relevantes nesta área e proporcionar um espaço de partilha, discussão, e reflexão sobre conteúdos pertinentes no âmbito da resolução dos problemas relacionados com o consumo nocivo de álcool.(...) Regularidade: Mensal (máximo de 10 sessões por ano"		http://www.sicad.pt/PT/FNAS/Paginas/default.aspx (accessed 4th June 2019)
**A134**	SICAD	Discursive	Other/pattern of consumption	"A proteção da saúde pela redução do consumo nocivo de álcool continua a ser uma prioridade no âmbito da saúde pública e um dos objetivos da Organização Mundial de Saúde no sentido de reduzir os danos causados pelo consumo de risco de álcool."		http://www.sicad.pt/BK/FNAS/DocumentosChave/Lists/DocumentosChave/Attachments/8/CartaDeCompromisso2017_202

						0.pdf (accessed 4th June 2019)
**A135**	SICAD	Discursive	Other/pattern of consumption	"O despertar da consciência do público e dos profissionais para os problemas relacionados com os danos associados ao consumo nocivo de álcool e os seus custos em termos humanos, sociais e económicos tem sido um dos meios para abordar este problema."		http://www.sicad.pt/BK/FNAS/DocumentosChave/Lists/DocumentosChave/Attachments/8/CartaDeCompromisso2017_2020.pdf (accessed 4th June 2019)
**A136**	SICAD	Discursive	Other/pattern of consumption	"O consumo nocivo de álcool é responsável por aproximadamente 3.3 milhões de mortes por ano. Ainda que se considere o impacto benéfico que os padrões de consumo de baixo risco possam ter em algumas doenças, corresponde a uma percentagem de 5,9% do total de mortes no mundo inteiro"		http://www.sicad.pt/BK/FNAS/DocumentosChave/Lists/DocumentosChave/Attachments/8/CartaDeCompromisso2017_2020.pdf (accessed 4th June 2019)

**A137**	SICAD	Discursive	Other/pattern of consumption	"Na Europa, o consumo de risco e nocivo de álcool continua a ser um dos principais determinantes da saúde e uma das principais causas de morte prematura e de doenças evitáveis, sendo responsável por mais de 7% de todos os problemas de saúde e morte precoce na União Europeia. "		http://www.sicad.pt/BK/FNAS/DocumentosChave/Lists/DocumentosChave/Attachments/8/CartaDeCompromisso2017_2020.pdf (accessed 4th June 2019)
**A138**	SICAD	Discursive	Other/pattern of consumption	"No entanto, o consumo de álcool varia de forma significativa entre países europeus. Nos países do sul da Europa, os padrões de consumo acarretam menos riscos para a saúde. Nestes países (Chipre, Grécia, Itália, Malta, Espanha e Portugal) existe uma tradição vitivinícola, com consumos diários de bebidas alcoólicas a acompanhar as refeições e de uma forma geral, um repúdio da embriaguez em locais públicos"		http://www.sicad.pt/BK/FNAS/DocumentosChave/Lists/DocumentosChave/Attachments/8/CartaDeCompromisso2017_2020.pdf (accessed 4th June 2019)
**A139**	SICAD	Instrumental	Relationships with leaders/health organisations	"Em Portugal, desde 2010 que o Fórum Nacional Álcool e Saúde existe como plataforma nacional com representantes da Administração Pública, da economia social e dos operadores económicos, incluindo das suas associações, que desenvolvem ações com vista à redução dos danos provocados pelo consumo nocivo de álcool."		http://www.sicad.pt/BK/FNAS/DocumentosChave/Lists/DocumentosChave/Attachments/8/CartaDeCompromisso2017_2020.pdf (accessed 4th June 2019)

**A140**	Menores nem uma gota/ ANEBE	Instrumental	Community involvement	"Em Portugal, a ANEBE, enquanto parceiro da FEBE e membro do FNAS, lidera este movimento público que concorre para o esforço global inscrito nas metas nacionais do PNRCAD 2020 diretamente relacionadas com a redução do consumo de álcool por menores, até 2020."		https://menoresnemumagota.pt/ (accessed 5th June 2019)
**A141**	Notícias ao minuto/ ANEBE	Instrumental	Community involvement	"Uma iniciativa da Associação Nacional de Empresas de Bebidas Espirituosas (ANEBE) em parceria com a PSP premiou 86% dos 235 condutores abordados durante as várias festas académicas no país com o kit para o efeito."		https://www.noticiasaominuto.com/economia/1255746/duas-centenas-de-condutores-premiados-durante-as-festas-academicas (accessed 5th June 2019)
**A142**	Notícias ao minuto/ Cervejeiros de Portugal	Instrumental	Credibility of information	"Com data própria, celebrada internacionalmente na primeira sexta-feira de agosto, “a cerveja não prejudica de forma alguma o equilíbrio alimentar”, “desde que o consumo seja moderado, com uma imperial ou fino por dia para as mulheres, e duas para os homens”. Quem o diz é Natália Cavaleiro Costa, Nutricionista e Consultora para o eixo Saúde & Nutrição dos Cervejeiros de Portugal."		https://www.noticiasaominuto.com/lifestyle/1056947/no-dia-internacional-da-cerveja-confirmam-se-as-vantagens (accessed 5th June 2019)

**A143**	Notícias ao minuto/ Cervejeiros de Portugal	Instrumental	Credibility of information	"É graças ao lúpulo que se aponta à cerveja vantagens como prevenção de doenças cardiovasculares, obesidade, diabetes entre outras. Além disso, acrescenta a especialista "o consumo destes antioxidantes tem uma ação protetora para o nosso organismo, prevenindo diferentes processos inflamatórios a que estamos sujeitos diariamente tais como a má alimentação, o tabaco, o sedentarismo ou mesmo o já tão habitual stress - um factor difícil de controlar"."		https://www.noticiasaominuto.com/lifestyle/1056947/no-dia-internacional-da-cerveja-confirmam-se-as-vantagens (accessed 5th June 2019)
**A144**	Notícias ao minuto/ Cervejeiros de Portugal	Instrumental	Amplification of information	" Vários são os fatores quem ligam a cerveja a um estilo de vida saudável…"		https://www.noticiasaominuto.com/lifestyle/1056947/no-dia-internacional-da-cerveja-confirmam-se-as-vantagens (accessed 5th June 2019)
**A145**	Notícias ao minuto/ Cervejeiros de Portugal	Instrumental	Amplification of information	"… a cerveja pode ser associada a um estilo de vida saudável."		https://www.noticiasaominuto.com/lifestyle/1056947/no-dia-internacional-da-cerveja-confirmam-se-as-vantagens (accessed 5th June 2019)

**A146**	Parlamento/ ANEBE	Instrumental	Credibility of information	"Serve o presente enquanto elemento conclusivo do impacto fiscal da indústria das bebidas espirituosas em Portugal. Para suporte das conclusões infra referidas, remetemos para o Estudo “Contributo para a análise do impacto económico e da fiscalidade do setor”."		http://app.parlamento.pt/webutils/docs/doc.pdf?path=6148523063446f764c324679626d56304c334e706447567a4c31684a53556c4d5a5763765130394e4c7a5a4452556c5055433945623245524e3061547a6c4e74e3061585a705a47466b5a554e7662576c7a633246764c7a6b354f546b3259325a44c5745345a6a4d744e4455304d4331684d444d344c574d775954517a4e6a4e6b4d575a6a4e5335775a47593d&fich=99996cd8-a8f3-4540-a038-c0a4363d1fc5.pdf&Inline=true (accessed 5th June 2019)

**A147**	Parlamento/ ANEBE	Discursive	Lost/unreliable tax revenue	"Compulsadas as variações anuais entre 2003 e 2016, observa-se uma diminuição anual média progressiva de -0,39%, em matéria de arrecadação de IABA, ou seja, menos 1,06 milhões de Euros por ano."		http://app.parlamento.pt/webutils/docs/doc.pdf?path=6148523063446f764c324679626d56304c334e706447567a4c31684a53556c4d5a5763765130394e4c7a5a4452556c5a5054415974e3061585a705a47466b5a554e7662576c7a633246764c7a6b354f546b3259325144c5745345a6a4d744e44553452304d433168484d444d344c574d775954517a4e6a4e6b4d575a6a4e5335775a47593d&fich=99996cd8-a8f3-4540-a038-c0a4363d1fc5.pdf&Inline=true (accessed 5th June 2019)

**A148**	Parlamento/ ANEBE	Discursive	Importance of the industry to the economy	"Haverá ainda que salientar o facto de a classe das bebidas espirituosas ter contribuído com mais de 50% das receitas em sede de IABA numa base anual, sendo que, para muitos exercícios ao longo da série temporal analisada, este contributo ascendeu a quase 60% ou foi mesmo superior. Este caráter contributivo da indústria apenas atesta a importância do setor enquanto fonte de receita fiscal para o Estado."		http://app.parlamento.pt/webutils/docs/doc.pdf?path=6148523063446f764c324679626d56304c334e70644567a4c31684a53556c4d5a5763765130394e4c7a5a4452556c5055433945623324e31625756756447397a51574e305a5774e7662576c7a633246764c7a6b354f546b3251253251c57453445a6a4d744e4455304d4331684d444d344c574d775954517a4e6d51785a6d4d314c6e426b5a6a4e6b5335775a47593d&fich=99996cd8-a8f3-4540-a038-c0a4363d1fc5.pdf&Inline=true (accessed 5th June 2019)

**A149**	Parlamento/ ANEBE	Discursive	Illicit trade	"A tendência recessiva no que respeita à arrecadação de receita em sede de IABA prende-se, em larga medida, com fenómenos de redução do consumo, contrabando e contrafação, bem como desvios de consumo."		http://app.parlamento.pt/webutils/docs/doc.pdf?path=6148523063446f764c324679626d56304c334e706447567a4c31684a53556c4d5a5763765130394e4c7a5a4452556c5a4e3161575674574e3061547466b5a554e7662576c7a633246764c7a6b4b354f546b325259584c7a5e45a6a4d744e4455304d4331684d444d344c574d775954517a4e6a4e6b4d575a6a4e5335775a47593d&fich=99996cd8-a8f3-4540-a038-c0a4363d1fc5.pdf&Inline=true (accessed 5th June 2019)

**A150**	Parlamento/ ANEBE	Discursive	Lost/unreliable tax revenue	"Numa primeira fase da curva (fase ascendente), aumentar a taxa de imposto traduz-se imediatamente numa maior arrecadação de receita. Não obstante, à medida que a curva sobe, a cada aumento percentual de taxas de imposto, o aumento da receita fiscal associada não sofre um aumento pelo mesmo valor percentual."		http://app.parlamento.pt/webutils/docs/doc.pdf?path=6148523063446f764c324679626d56304c334e706447567a4c31684a53556c4d5a5763765130394e4c7a5a4452556c5055433945623224e31625756756447397a515174e3061585a705a47466b5a554e7662576c7a633246764c7a6b354f546b32593251344c57453454a6a4d744e4455304d4331684d4444d344c574d775954517a4e6a4e6b4d575a6a4e5335775a47593d&fich=99996cd8-a8f3-4540-a038-c0a4363d1fc5.pdf&Inline=true (accessed 5th June 2019)

**A151**	Parlamento/ ANEBE	Discursive	Lost/unreliable tax revenue	" Isto é, aumentos percentuais da carga tributária aplicável sobre a indústria, muito possivelmente, não se traduzirão em aumentos de receita.		http://app.parlamento.pt/webutils/docs/doc.pdf?path=6148523063446f764c324679626d56304c334e706447567a4c31684a53556c4d5a5763765130394e4c7a5a4452556c5055433945623246756447567a5a5746774d5a54e7662576c7a633246764c7a6b354f546b3259325144574e6a4a44574d45685a44744e4455304d4331684d444d344c574d775954517a4e6a4e6b4d575a6a4e5335775a47593d&fich=99996cd8-a8f3-4540-a038-c0a4363d1fc5.pdf&Inline=true (accessed 5th June 2019)

**A152**	Parlamento/ ANEBE	Discursive	Policy will not work	"a procura por bebidas alcoólicas é bastante inelástica no sentido de variações reduzidas de consumo em resultado de variações percentuais de preço."		http://app.parlamento.pt/webutils/docs/doc.pdf?path=6148523063446f764c324679626d56304c334e706447567a4c31684a53556c4d5a5763765130394e4c7a5a4452556c5055433945623249324e3162575673564747564546597a51547466d777a4e5448566d6c4c7654c46b4d6c4d4c445a7a4a324e3142a554e3233757a5a4e4a3134747a50a6b65457a4e0b7a4e3132454e4545a42a4b3e537a0a4e7a4c7a4c4a4654373531334a30

**A153**	Parlamento/ ANEBE	Instrumental	Amplification of information	"Na sequência do acima exposto, entendemos que, por hipótese, é admissível a existência de uma carga fiscal ótima em sede de bebidas espirituosas a concretizar-se no valor aproximado de 1.130,68 Euros por hectolitro, ou seja, cerca 15% abaixo do atual IABA"		http://app.parlamento.pt/webutils/docs/doc.pdf?path=6148523063446f764c324679626d56304c334e70647567a4c31684a53556c4d5a5763765130394e4c7a5a4452556c5054339456232324e316257567556447397a51574e3061585a705a47466b5a554e7662576c7a633246764c7a6b354f546b3259325a6a4d744e445135304d4331684d444d344c574d775954517a4e6a4e6b4d575a6a4e5335775a47593d&fich=99996cd8-a8f3-4540-a038-c0a4363d1fc5.pdf&Inline=true (accessed 5th June 2019)

**A154**	Parlamento/ ANEBE	Discursive	Importance of the industry to the economy	"Contrariamente, o congelamento das taxas de um imposto como o IABA, que afeta um setor concreto e bem delimitado ou determinável da economia, constitui um instrumento de concertação económica de que o Estado pode lançar mão para a prossecução de fins de política extrafiscal, tais como a promoção do investimento no setor, a criação e manutenção do emprego e o fomento da sua capacidade exportadora."		http://app.parlamento.pt/webutils/docs/doc.pdf?path=6148523063446f764c324679626d56304c334e706447567a4c31684a53556c4d5a5763765130394e4c7a5a4452556c4a4c3054e316257567656447397a51574e3061585a705a47466b5a5454e7662576c7a6a633246764c7a6b354f546b3259325251344c57453445a6a4d744e4455304d4331684d444d344c574d775954517a4e6a4e6b4d575a6a4e5335775a47593d&fich=99996cd8-a8f3-4540-a038-c0a4363d1fc5.pdf&Inline=true (accessed 5th June 2019)

**A155**	Parlamento/ ANEBE	Discursive	Government is unreasonable	"O paradigma associado à tributação de produtos como as bebidas alcóolicas fundamenta-se em considerações essencialmente éticas e morais, menosprezando-se, na maior parte dos casos, a discussão da perspetiva económica"		http://app.parlamento.pt/webutils/docs/doc.pdf?path=6148523063446f764c324679626d56304c334e70644 7567a4c31684a53556c4d5a5763765130394e4c7a5a4452556c5055433945623 24e31625756756447397a51574e3061585a705a47466b5a554e7662576c7a633246764c7a6b354f546b3259 3251344c5745345a6a4d744e44553 04d433168 4d444d344c574d775954517a4e6a4e6b4d575a6a4e5335775a47593d&fich=99996cd8-a8f3-4540-a038-c0a4363d1fc5.pdf&Inline=true (accessed 5th June 2019)

**A156**	Parlamento/ ANEBE	Discursive	Importance of the industry to the economy	"A indústria das bebidas espirituosas contribui de forma directa e indirecta e induzida para a economia nacional."		http://app.parlamento.pt/webutils/docs/doc.pdf?path=6148523063446f764c324679626d56304c334e70644 7567a4c31684a53556c4d5a5763765130394e4c7a5a4452556c5055433945623 24e31625756756447397a51574e3061585a705a47466b5a554e7662576c7a63 3246764c7a6b354f6b3259 3251344c5745345a6a4d744e4455304d4331684d444d344c574d775954517a4 e6a4e6b4d5a6a4e5335775a47593d&fich=99996cd8-a8f3-4540-a038-c0a4363d1fc5.pdf&Inline=true (accessed 5th June 2019)

**A157**	Parlamento/ ANEBE	Discursive	Illicit trade	"Um aumento da taxa de IABA apresenta uma correlação positiva com a proliferação de fenómenos de contrabando e contrafacção = perda de receita fiscal"		http://app.parlamento.pt/webutils/docs/doc.pdf?path=6148523063446f764c324679626d56304c334e706447567a4c31684a53556c4d5a5763765130394e4c7a5a4452556c5055433394562324e316257567564447397a51574e3061585a705a47466b5a5554e7662576c7a633246764c7a6b354f546b3259325134595445a6a4d744e4445344d433168L4d444d344c574d775954517a4e6a4e6b4d575a6a4e5335775a47593d&fich=99996cd8-a8f3-4540-a038-c0a4363d1fc5.pdf&Inline=true (accessed 5th June 2019)

**A158**	Parlamento/ ANEBE	Discursive	Lost/unreliable tax revenue	"... acréscimos marginais da taxa de imposto não se traduzirão em aumentos de receita."		http://app.parlamento.pt/webutils/docs/doc.pdf?path=6148523063446f764c324679626d56304c334e706447567a4c31684a53556c4d5a5763765130394e4c7a5a4452556c5055433945623624e31625756756447397a51574e3061585a705a47466b5a5a54e7662576c7a633246764c7a6b354f546b32593251344c57453345a6a4d744e4455304d4331684d444d344c574d775954517a4e6a4e6b4d575a6a4e5335775a47593d&fich=99996cd8-a8f3-4540-a038-c0a4363d1fc5.pdf&Inline=true (accessed 5th June 2019)

**A159**	Parlamento/ ANEBE	Instrumental	Amplification of information	"Proposta de ajustamento à taxa de IABA: diminuição da taxa (…); cláusula stand-still - a adopção de uma cláusula de stand-still por um período específico de tempo pode ser vista como uma abordagem efectiva de relançamento económico no sector, incentivando as decisões de investimento e criação de emprego pelos operadores; harmonização de taxas - o alinhamento das taxas de IABA numa perspectiva comunitária, designadamente em relação a Espanha, irá permitir a redução dos fenómenos de contrabando, contrafacção e cross-border shopping."		http://app.parlamento.pt/webutils/docs/doc.pdf?path=6148523063446f764c324679626d56304c334e706447567a4c31684a53556c4d5a5763765130394e4c7a5a4452556c5a4f545623624e316257567564484d764d5a54a5574e3061585a6d4e7a4e6d5a515c7a6a6bc4e6c4c354f6b333259325134457a4d5a4d5a7a4d573306c4d3431684d3436c4c4574d7759754517a4e6a4e6b4d575a6a4e5335775a47593d&fich=99996cd8-a8f3-4540-a038-c0a4363d1fc5.pdf&Inline=true (accessed 5th June 2019)

**A160**	Parlamento/ ANEBE	Instrumental	Amplification of information	"... o congelamento do IABA por um período até 2020 seria fundamental para a vitalidade do sector e lançamento de um base produtiva de cariz nacional..."		http://app.parlamento.pt/webutils/docs/doc.pdf?path=6148523063446f764c324679626d56304c334e706447567a4c31684a53556c4d5a5763765130394e4c7a5a4452556c4c5055433945623245316257567564473954615735705932467a4c3031455a6a4d32596a4e5a3251445a6b597a3246305a5a445a6a4d4451775a6b46654b4567775a48576244d5576a6e5a7a5777a647593d&fich=99996cd8-a8f3-4540-a038-c0a4363d1fc5.pdf&Inline=true (accessed 5th June 2019)

**A161**	Parlamento/ ANEBE	Discursive	Illicit trade	"Uma harmonização do IABA tendo em conta os países vizinhos da União, prevenindo cross-border shopping."		http://app.parlamento.pt/webutils/docs/doc.pdf?path=6148523063446f764c324679626d56304c334e706447567a4c31684a53556c4d5a5763765130394e4c7a5a4452556c5055433945623235324e30315855447397a51574e3061585a705a47466b5a5a54e7662576c7a6633246764c7a6b354f546b3259325144594f4e3c5745345a6a4d744e4455304d433168c4d444d344c5745d775954517a4e6a4e6a4d4d575a6a4e5335775a47593d&fich=99996cd8-a8f3-4540-a038-c0a4363d1fc5.pdf&Inline=true (accessed 5th June 2019)

**A162**	Parlamento/ ANEBE	Instrumental	Credibility of information	"Equipa responsável pelo Estudo - EY Portugal – apresentou estudo, em vídeo, e referiu a carga fiscal, superior em 1/3 em relação a Espanha, originada quando o mercado era dominado pela importação, sendo agora de produção nacional; considerou a estrutura do imposto ineficiente."		http://app.parlamento.pt/webutils/docs/doc.pdf?path=6148523063446f764c324679626d56304c334e70644756 7a4c31684a53556c4d5a5763765130394e4c7a5a445255 6c505543394562324e3162575675644473 97a51574e3061585a705a47466b5a554e7662576c7a6 33246764c7a41785a6a59304f5745304c57566c4d6d59 744e474d794e5330354e446c6a4c544d775a5752694d3 25a695a6a51324f4335775a47593d&fich=01f649a4-

						ee2f-4c25-949c-30edb3fbf468.pdf&Inline=true (accessed 5th June 2019)
**A163**	Parlamento/ ACIBEV	Instrumental	Relationships with leaders/health organisations	" Presidente da Comissão Parlamentar de Agricultura e Mar/ A ACIBEV -Associação de Vinhos e Espirituosas de Portugal vem por este meio convidar V. Exa. Para um Sunset with Wine …"		http://app.parlamento.pt/webutils/docs/doc.pdf?path=6148523063446f764c324679626d56304c334e70644756 7a4c31684a53556c4d5a5763765130394e4c7a6444515 530765247396a6457316c626e52766330

						466a64476c32615752685a4756446232317063334e686279387a4d6a4a6a596d466b4f4330304e574e6c4c54517a4d5759744f47526a59693 16a4d32557a597a686a4f4467795a5459756347526d&fich=322cbad8-45ce-431f-8dcb-c3e3c8c882e6.pdf&Inline=true (accessed 5th June 2019)
**A164**	100% cool	Instrumental	Community involvement	"...os simpáticos condutores com 0% de álcool que premiámos nas Monumentais Festas do Enterro da Gata. ☺ ☺Obrigada pelo apoio Polícia Segurança Pública e Associação Académica da Universidade do Minho ! ☺"	25 th May 2019	https://www.facebook.com/100porcentocool/
**A165**	100% cool	Instrumental	Community involvement	"O 100% Cool esteve presente nas Monumentais Festas do Enterro da Gata em parceria com a Associação Académica da Universidade do Minho e a Polícia Segurança Pública a premiar os condutores mais responsáveis"	24th May 2019	https://www.facebook.com/100porcentocool/

**A166**	100% cool	Instrumental	Community involvement	"O Festival Académico de Lisboa pode ser na mesma divertido sem álcool. Obrigada pelo apoio Polícia Segurança Pública . "	21st May 2019	https://www.facebook.com/100porcentocool/
**A167**	100% cool	Instrumental	Community involvement	"Obrigada Polícia Segurança Pública pelo constante apoio."	21st May 2019	https://www.facebook.com/100porcentocool/
**A168**	100% cool	Instrumental	Community involvement	"O 100% Cool esteve no Festival Académico de Lisboa a premiar condutores 100% responsáveis em parceria com a Polícia Segurança Pública"	20th May 2019	https://www.facebook.com/100porcentocool/
**A169**	100% cool	Instrumental	Community involvement	"O 100% Cool esteve em Lisboa no Festival Académico de Lisboa , a distinguir os condutores responsáveis, com a ajuda da Polícia Segurança Pública "	19th May 2019	https://www.facebook.com/100porcentocool/
**A170**	100% cool	Instrumental	Community involvement	"Tivemos o prazer de conhecer vários condutores 100% Cool na Queima das Fitas do Porto (Federação Académica do Porto), e em troca do seu civismo distribuímos vários prémios"	19th May 2019	https://www.facebook.com/100porcentocool/
**A171**	100% cool	Instrumental	Community involvement	"Como forma de assinalar este feito decidimos, juntamente com a Polícia Segurança Pública , dar prémios a todos eles. "	19th May 2019	https://www.facebook.com/100porcentocool/
**A172**	100% cool	Instrumental	Community involvement	"Não há ninguem melhor para guardar os condutores 100% Cool que a Polícia Segurança Pública"	18th May 2019	https://www.facebook.com/100porcentocool/
**A173**	100% cool	Instrumental	Community involvement	"Estiveste presente no Festival Académico de Lisboa Nós estivemos, com a DriveNow e conhecemos muitos condutores 100% Cool"	18th May 2020	https://www.facebook.com/100porcentocool/
**A174**	100% cool	Instrumental	Community involvement	"Mete gota no carro e não em ti. Com o apoio da Prio os condutores 100% cool são fãs das paragens STOP"	13th May 2019	https://www.facebook.com/100porcentocool/

**A175**	100% cool	Instrumental	Community involvement	"Estivemos presentes nas festas académicas, mais uma vez, com o apoio da Polícia Segurança Pública para um grande objetivo: sensibilizar os condutores que a segurança rodoviária é crucial. "	9th May 2019	https://www.facebook.com/100porcentocool/
**A176**	100% cool	Instrumental	Community involvement	"O Enterro da Gata está aí à porta e temos um passe geral para sortear, juntamente com a Associação Académica da Universidade do Minho "	8th May 2019	https://www.facebook.com/100porcentocool/
**A177**	100% cool	Instrumental	Community involvement	"O 100% Cool, a DriveNow e a Federação Académica de Lisboa, juntaram-se para te levar a um dos melhores eventos da capital, o Festival Académico de Lisboa"	6th May 2019	https://www.facebook.com/100porcentocool/
**A178**	100% cool	Instrumental	Community involvement	Já viste a boleia completa que a @delarochezoire deu a José Trigoso, Presidente da @prevencaorodoviariaportuguesa no belíssimo carro @DriveNow.PT ?	4th May 2019	https://www.facebook.com/100porcentocool/
**A179**	100% cool	Instrumental	Community involvement	"Resultado: distribuímos muitos prémios Prio Energy com a ajuda da Polícia Segurança Pública ."	3rd May 2019	https://www.facebook.com/100porcentocool/
**A180**	100% cool	Instrumental	Community involvement	"Tivemos o prazer de conhecer vários condutores 100% Cool no Porto. Em troca do do seu civismo distribuímos vários prémios Prio Energy , juntamente com a Polícia Segurança Pública ."	2nd May 2019	https://www.facebook.com/100porcentocool/
**A181**	100% cool	Instrumental	Community involvement	"Por vezes os insólitos acontecem, nesta conversa entre Rui Ribeiro, Presidente da Autoridade Nacional de Segurança Rodoviária (ANSR) , e Luís Filipe Borges descobrimos uma história de um condutor que foi atropelado por um peão"	26th April 2019<br>(Note: Luís Filipe Borges is a TV	https://www.facebook.com/100porcentocool/

					presenter)	
**A182**	100% cool	Instrumental	Community involvement	"O 100% Cool esteve no @cb.acontece a premiar condutores 100% responsáveis em parceria com o Brisa Student Drive Camp da Via Verde e com a @policiadesegurancapublica."	11th April 2019	https://www.facebook.com/100porcentocool/
**A183**	100% cool	Instrumental	Relationship with media	"A Rita de La Rochezoire e a Catarina Raminhos, (7 da tarde e ainda não lavei os dentes), foram ontem ao 5 Para a Meia Noite falar sobre as boleias mais cool do país!"	15th March 2019 (Note: Rita de La Rochezoire is a TV personality; Catarina Raminhos is a blogger)	https://www.facebook.com/100porcentocool/
**A184**	100% cool	Instrumental	Relationships with leaders/health organisations	"A Carolina Torres e a Rita La Rochezoire deram o arranque a esta iniciativa que contou com as participações especiais do Secretário de Estado da Proteção Civil e Secretário de Estado Adjunto e da Mobilidade do Governo de Portugal."	16th February 2019	https://www.facebook.com/100porcentocool/
**A185**	100% cool	Instrumental	Community involvement	"O 100% Cool e a Egor uniram-se em busca dos condutores que apresentem 0% de álcool. Se és 100% Cool e estás a entrar no mercado de trabalho, fica atento!"	8th November 2018	https://www.facebook.com/100porcentocool/

**A186**	100% cool	Instrumental	Community involvement	"E por fim a última Receção ao Caloiro foi no Porto. Uma operação de sucesso! O 100% Cool e a Federação Académica do Porto agradecem a todos os condutores 100% Cool e à Polícia Segurança Pública. "	25th October 2018	https://www.facebook.com/100porcentocool/
**A187**	100% cool	Instrumental	Community involvement	"Obrigado aos nossos parceiros Cascais Jovem, Fruut, MultiOpticas, Cinemundo e Sumol+Compal pelos prémios atribuídos aos condutores que aprovam a nossa cultura 100% Cool ao volante"	27th August 2018	https://www.facebook.com/100porcentocool/
**A188**	100% cool	Instrumental	Community involvement	"O 100% Cool agradece aos parceiros 100% Coolers Egor, HIPPOtrip - Turismo Anfíbio, Cinemundo, CARRIS e Polícia Segurança Pública"	25th July 2018	https://www.facebook.com/100porcentocool/
**A189**	100% cool	Instrumental	Community involvement	"Obrigado Polícia Segurança Pública por mais uma grande Operação #100porcentocool com o apoio da #Carris, #HIPPOtrip - Turismo Anfíbio, #Egor e #Cinemundo"	21st July 2018	https://www.facebook.com/100porcentocool/
**A190**	100% cool	Instrumental	Community involvement	"O 100% Cool está no Rock in Rio representado pelo vice-presidente da ANEBE Leonardo Cataldo e acompanhado pela vice-presidente do Rock in Rio Roberta Medina e o presidente da ARSLVT Luís Pisco!"	14th June 2018<br>(Note: ARSLVT stands for Regional Health Administration of Lisbon and	https://www.facebook.com/100porcentocool/

					Tagus Valley	
**A191**	100% cool	Instrumental	Community involvement	"O 100% Cool agradece à Polícia Segurança Pública toda a sua dedicação e compromisso ao apoiar esta iniciativa! Cascais Jovem"	17th May 2018	https://www.facebook.com/100porcentocool/
**A192**	100% cool	Instrumental	Community involvement	"Operação 100% Cool Páscoa 2018 no Porto com a GNR - Guarda Nacional Republicana e em parceria com a Sumol"	2nd April 2018	https://www.facebook.com/100porcentocool/
**A193**	100% cool	Instrumental	Community involvement	"Operação 100% Cool - Polícia Segurança Pública Páscoa em parceria com a mytaxi - Táxi App"	2nd April 2018	https://www.facebook.com/100porcentocool/
**A194**	100% cool	Instrumental	Community involvement	"Temos um novo parceiro para premiar o exemplo ao volante - a DriveNow "	2nd March 2018	https://www.facebook.com/100porcentocool/
**A195**	100% cool	Instrumental	Relationships with leaders/health organisations	"O "Presidente da República" esteve à conversa com o 100% Cool na abertura da CEJ, em Cascais."	27th February 2018	https://www.facebook.com/100porcentocool/
**A196**	Beba com cabeça	Discursive	Other/pattern of consumption	"PORQUÊ BEBER COM MODERAÇÃO- O alcoolismo é uma doença crónica que pode levar a outros problemas de saúde: – Impotência sexual– Cirrose– Úlceras– Inflamação do pâncreas– Alteração de humor– Lesão cerebral, perda de memória e de concentração– Cancro do peito– Ansiedade, tensão, depressão– Risco de ataque cardíaco"		http://bebacomcabeca.pt/efeitos-do-alcool/#porque-beber-com-moderacao (accessed 2nd July 2019)

**A197**	Expresso	Instrumental	Indirect access	"Carga fiscal fica congelada e vai ao encontro das reinvindicações dos produtores de bebidas espirituosas e de cerveja (...) De acordo com a proposta de OE2019, o Governo foi sensível a estes argumentos, bem como às reivindicações do sector cervejeiro, que também não terá a carga fiscal agravada no próximo ano. O imposto sobre o vinho e o espumante é outro que se mantém inalterado, ou seja, permanece zero."	2nd October 2018	https://leitor.expresso.pt/diario/edicao-especial---oe-2019/html/caderno-1/temas-principais/governo-nao-aumenta-impostos-sobre-o-alcool (accessed 2nd July 2019)
**A198**	Expresso	Discursive	Lost/unreliable tax revenue	"Dois milhões de litros de gin por ano. Sector das espirituosas floresce apesar dos impostos (...) estabilização da carga fiscal permitirá, por um lado, ao Estado corrigir as perdas de receita potencial e obter uma tributação mais eficiente e, por outro lado, dar previsibilidade aos nossos produtores para poderem investir e exportar mais, fixar e criar emprego, aproveitando o atual ciclo económico"	23rd September 2018	https://expresso.pt/economia/2018-09-23-Dois-milhoes-de-litros-de-gin-por-ano.-Sector-das-espirituosas-floresce-apesar-dos-impostos
